# *EARLY ABORTION 1* is an evolutionarily conserved gene required for plant reproduction

**DOI:** 10.1093/jxb/erag142

**Published:** 2026-03-23

**Authors:** Jingjing Zhou, Wei Wang, Li Zhang, Ylva Bruce, Shaochun Zhu, André Mateus, Totte Niittylä

**Affiliations:** Umeå Plant Science Centre, Department of Forest Genetics and Plant Physiology, Swedish University of Agricultural Sciences, Umeå 901 83, Sweden; Umeå Plant Science Centre, Department of Forest Genetics and Plant Physiology, Swedish University of Agricultural Sciences, Umeå 901 83, Sweden; Yazhouwan National Laboratory, Sanya, Hainan 572025, China; Yazhouwan National Laboratory, Sanya, Hainan 572025, China; Umeå Plant Science Centre, Department of Forest Genetics and Plant Physiology, Swedish University of Agricultural Sciences, Umeå 901 83, Sweden; Department of Chemistry, Umeå University, Umeå 90736, Sweden; The Laboratory for Molecular Infection Medicine Sweden (MIMS), Umeå, Sweden; Department of Chemistry, Umeå University, Umeå 90736, Sweden; The Laboratory for Molecular Infection Medicine Sweden (MIMS), Umeå, Sweden; Umeå Plant Science Centre, Department of Forest Genetics and Plant Physiology, Swedish University of Agricultural Sciences, Umeå 901 83, Sweden; Ohio State University, USA

**Keywords:** Arabidopsis, gametophyte, seed development

## Abstract

The functions of approximately one-third of the proteins in the model plant Arabidopsis remain unknown. It is likely that some of the genes encoding these proteins are essential, and thus indispensable for the survival of the plant; furthermore, these genes would be included in the minimum viable set required for plant life. Evolutionarily conserved single copy genes in flowering plants are enriched in essential housekeeping functions. Building on this observation, we designed a reverse genetic screen that focuses on evolutionarily conserved single copy Arabidopsis genes of unknown function with predominant expression in meristematic cells. This approach identified a previously uncharacterized essential Arabidopsis gene, named as *EARLY ABORTION 1* (*EBO1*). Mutation of the *EBO1* locus disrupts gametophyte and/or early embryo development, resulting in defective ovule or seed development. A functional fluorescent EBO1 fusion protein was found to localize to the nucleus, and co-immunoprecipitation experiments detected an interaction between EBO1 and Nucleolar Protein 58 (NOP58) and proteins involved in RNA metabolism, chromatin modification, and transcription. The presented results open a new line of investigation into an evolutionarily conserved mechanism involved in the development of both male and female gametophytes as well as seeds.

## Teaser

An essential Arabidopsis gene was identified and named *EARLY ABORTION 1* (*EBO1*); its mutation disrupts gametophyte and/or embryo development, and its protein localizes to the nucleus.

## Introduction

Many essential genes encode proteins of unknown function (PUFs). Several independent large-scale genetic studies in the model plant Arabidopsis have identified mutations in genes with essential roles in basic cellular processes. These mutations typically result in phenotypes that include seed, zygote, or embryo defects (McElver *et al*., 2001; Meinke *et al*., 2008, 2009; Lloyd and Meinke, 2012; Meinke, 2020). Approximately 5% (27 out of 510) of the essential Arabidopsis genes identified so far encode PUFs. It is likely that more essential genes await discovery amongst the currently 4599 Arabidopsis genes annotated as unknown molecular function involved in an unknown biological process. It is important to investigate these genes to elucidate new biological processes and discover new areas of research. Functional genomic investigations of genes and proteins with completely unknown functions can be challenging, with a focus on single-copy genes potentially alleviating some of these challenges.

Most genes within plant genomes belong to a defined gene family based on sequence similarity; nevertheless, these genomes also contain many single-copy genes that seem to be under selective pressure to remain solitary. The origins of gene families can be traced back to gene and whole-genome duplication events (Blanc and Wolfe, 2004; Van de Peer *et al*., 2009), which, along with the smaller-scale gene duplications, play a crucial role in providing new genetic material for the process of evolution (Panchy *et al*., 2016). However, comparative genome sequence analyses have revealed that certain genes are adversely affected by duplication. These genes, known as duplication-resistant genes, have independently reverted to a single-copy state in numerous plant species following duplication events (Paterson *et al*., 2006; Duarte *et al*., 2010; De Smet *et al*., 2013). Such single-copy genes often encode proteins that are detrimentally impacted by the presence of two or more copies of the underlying gene. These dynamics may be explained by factors such as gene dosage-linked sensitivity of gene transcript levels or dominant negative effects following a mutation in one copy of the gene; this is often the case for protein complexes (Veitia *et al*., 2008; De Smet *et al*., 2013). We selected single-copy gene PUFs in Arabidopsis as candidates in a reverse genetic screen with the aim of alleviating some of the gene redundancy and phenotyping challenges associated with PUF characterization. In our previous work the combination of publicly available gene expression data and simple seed abortion phenotyping identified the gene *OPENER*, which is involved in ribosome biogenesis (Wang *et al*., 2019, 2025). Here we applied the same technique, and report another single-copy Arabidopsis gene that is essential for plant reproduction.

## Materials and methods

### Plant materials and mutant isolation

All of the Arabidopsis plant materials used in this study represented the Columbia-0 accession. The *ebo1-1*/+ (*SALK_030411*) mutants were obtained from the Nottingham Arabidopsis Stock Centre (NASC, Nottingham, UK). The sequences of all the primers used in the study are listed in Supplementary Table S1. The *ebo1-2* mutant was generated using the CRISPR-cas9 DNA editing technique. More specifically, two single guide RNAs (CCAAAGAAAAATCATGGAA and AAACTTAAAGGCTTCTTGG) were cloned into the pHEE401E vector as previously described (Xing *et al.*, 2014; Wang *et al.*, 2015) to generate a deletion in the *EBO1* coding sequence. The seeds were pre-germinated on Murashige and Skoog (MS)-medium agar plates under conditions of 16 h light (∼100 μmol m^−2^ s^−1^)/8 h dark at 22 °C. The seedlings were later transplanted into soil for further growth under the same conditions. The T-DNA insertion site of *ebo1-1* was determined by PCR using the primer set LBb1.3/030411-LP2 (Supplementary Table S1), while the heterozygosity of the *ebo1-1* and *ebo1-2* mutations was determined by PCR using the primer set 030411-LP2/030411-RP2 (Supplementary Table S1). The primer set of U6-26-1DF/U6-29-1DR (Supplementary Table S1) was used to determine the existence of a CRISPR transgenic cassette in the *ebo1-2*/+ plants. The Mix2Seq service provided by Eurofins Genomics (Ebersberg, Germany) was utilized to determine the modified DNA sequence of the *ebo1-2* mutation.

### Phenotypic characterization of *ebo1* mutants

The siliques were photographed with a Leica MZ16 stereomicroscope (Leica, Wetzlar, Germany). Ovules and developing seeds were cleared in Hoyers solution (7.5 g gum arabic, 100 g chloral hydrate, and 5 ml glycerol in 30 ml water) as described by Meinke (1994), and photographed with a Zeiss AxionPlan 2 Imaging microscope (Zeiss, Oberkochen, Germany). Alexander staining and 4′,6-diamidino-2-phenylindole (DAPI) staining of pollen grains were performed as described by Alexander (1969), Dou *et al.* (2011), and Yang *et al.* (2009), and photographed with a Zeiss AxionPlan 2 Imaging microscope. Pollen germination *in vitro* and a pollen tube growth assay *in vivo* were performed as described by Jiang *et al.* (2005). Pollen germination *in vitro* was photographed with a Leica MZ16 stereomicroscope, and the *in vivo* pollen tubes were photographed with a Zeiss AxionPlan 2 Imaging microscope.

### Complementation of the *ebo1-1*/+ mutants and *EBO1* expression and sub-cellular localization

The *pEBO1::EBO1-YFP* construct was generated using the GreenGate cloning system (Lampropoulos *et al*., 2013). The 2 kb long *EBO1* promoter before the translation start site was cloned with the primer set EBO1-ggA-F/EBO1-ggA-R (Supplementary Table S1), and was assembled into module A. The *EBO1* coding sequence was cloned with the primer set EBO1-ggC-F/EBO1-ggC-R (Supplementary Table S1), and was assembled into module C. *mVenus-SPY* was assembled into module D, and the *UBQ10* terminator was assembled into module E to terminate expression of the *pEBO1::EBO1-YFP* cassette. pGGZ001 was the destination vector. The construct was transformed into *ebo1-1*/+ mutants using the floral dip method mediated by *Agrobacterium tumefaciens* (Clough and Bent, 1998). Yellow fluorescent protein (YFP) signals were observed and photographed with a confocal Zeiss LSM800 CLSM confocal microscope.

### Green fluorescent protein-trap immunoprecipitation and sample preparation

Total proteins from pRPS5A:YFP and pEBO1:EBO1-YFP plant calli were extracted with an extraction buffer [50 mM Tris, pH 7.5, 150 mM NaCl, 1 mM EDTA, 0.5% Triton X-100, 0.5% IGEPAL CA-630, 10% (v/v) glycerol, 1× Complete protease inhibitor cocktail (Roche, Basel, Switzerland)], and incubated with green fluorescent protein (GFP)-Trap-MA (ChromoTek, Martinsried, Germany) beads for 30 min at 4 °C. The beads were then washed five times with extraction buffer. Proteins were eluted from the beads with an elution buffer (0.1 M glycine, pH 2.5, 1% Triton X-100), which was pipetted over the beads for 30 s. Next, 5% v/v 1 M Tris base was added immediately to bring the pH to about 7.5. The eluted proteins were then digested into peptides using a modified SP3 protocol (Hughes *et al*., 2019). Briefly, SpeedBeads, that is, magnetic carboxylate-modified particles (Sigma Aldrich, St Louis, MO, USA; beads A hydrophylic, cat. no. GE45152105050250; beads B hydrophobic, cat. no. GE65152105050250) were combined at a ratio of 1:1 v/v and washed four times with LC-MS water. The beads were then mixed with each sample in binding buffer (50% ethanol and 2.5% formic acid), and incubated for 15 min at room temperature with shaking at 500 rpm. The beads were then transferred onto one filter plate (0.22 µm, Sigma Aldrich, cat. no. MSGVN2210). The unbound fraction was removed by centrifugation at 1000 rcf. The beads remained on the filter and were washed four times with 70% ethanol. Trypsin (0.2 µg) was then included in a digestion solution [100 mM HEPES, pH 7.5, 5 mM chloroacetamide, 1.2 mM Tris(2-carboxyethyl)phosphine], which was added to each sample on the plate. The samples underwent overnight digestion at room temperature with shaking at 500 rpm. The flowthrough containing peptides was collected with centrifugation at 1000 rcf, after which 10 µl of 2% dimethyl sulfoxide was added to the beads to elute any bound peptides; the eluant was pooled with the previous flowthrough. Peptides were desalted using an Oasis HLB plate (Waters, Milford, MA, USA; cat. no. 186001828BA) according the factory protocols, and then dried by speed vac. Three biological replicates were performed for the co-immunoprecipitation (co-IP) experiment.

### Mass spectrometry and data analysis

Dried peptides were dissolved with 20 µl 0.1% formic acid in water. A total of 5 µl peptides from each sample was introduced into a mass spectrometer using Vanquish Neo (Thermo Scientific, Waltham, MA, USA). The trapping column was a PEPMAP NEO C18 (5 µm particle size, 300 µm × 5 mm, Thermo Scientific), while the analytical column was a nanoEaseTM M/Z HSS C18 T3 (100 Å, 1.8 µm particle size, 75 µm × 250 mm, Waters). The run time for separation and elution was 90 min, which started with 98% mobile phase A (water and 0.1% formic acid) and 2% mobile phase B (80% acetonitrile and 0.1% formic acid), rising to 8% B over 4 min, to 27% B over 60 min, and then to 40% B in 13 min. Next, mobile phase B was increased to 80% in 0.1 min and was held constant for 4 min, after which B was decreased to 2% in 0.5 min; this was followed by column equilibration.

Data acquisition on Exploris 480 (Thermo Scientific) was carried out using a data-dependent method. Survey scans covering the mass range of 375–1500 *m*/*z* were acquired at a resolution of 120 000 (at *m*/*z* 200), RF lens of 40%, and normalized automatic gain control (AGC) of 300%. A maximum cycling time of 2 s was used to control the number of precursors for tandem mass spectrometry (MS/MS, MS2) analysis. Charge states included between two and six charges. Dynamic exclusion was set to exclude the previously selected precursors for 35 s. MS2 scans were acquired at a resolution of 15 000 (at *m*/*z* 200), with the AGC target value set to ‘auto’. The isolation window was 1.4 *m*/*z*. Higher-energy collisional dissociation fragmentation was induced with a normalized collision energy of 30. Isotopes were excluded for the MS2 analysis.

The obtained raw data were searched against the *Arabidopsis thaliana* UniProt FASTA using FragPipe (version 18), while label-free quantification was achieved using the LFQ-MBR workflow (Kong *et al*., 2017). Proteins identified from contaminants were removed. Moreover, only the proteins that were quantified from more than one replicate in each group were retained for further analysis. The intensity of the measured protein levels was analysed using Perseus (Tyanova *et al*., 2016) and presented as a volcano plot. Missing data were replaced from the normal distribution. The log10 fold change of protein intensity, shown on the *x*-axis of the volcano plot, indicates the extent to which a certain protein was enriched in EBO1–YFP co-IP data relative to the YFP control co-IP data. The −log10 *P*-values of differences between the two data sets are shown on the *y*-axis. The differences between data sets were calculated using Student’s *t*-test moderated by the Benjamini–Hochberg method. The false discovery rate was set to 0.05 and S0 was set to 1.

### Transient expression of proteins in tobacco and co-immunoprecipitation

Transient expression of p35S:EBO1-GFP/p35S:NOP58B-3xFLAG and p35S:GFP/p35S:NOP58B-3xFLAG in tobacco leaves was performed by agroinfiltration of tobacco leaves. Total proteins of p35S:EBO1-GFP/p35S:NOP58B-3xFLAG and p35S:GFP/p35S:NOP58B-3xFLAG were extracted from infiltrated leaves with extraction buffer [50 mM Tris, pH 7.5, 150 mM NaCl, 1 mM EDTA, 0.5% Triton X-100, 0.5% IGEPAL CA-630, 10% (v/v) glycerol, 1×Complete protease inhibitor cocktail (Roche)]. Total proteins were incubated with GFP-Trap-MA (Chromotek) beads for 1 h at 4 °C. The beads were washed three times with extraction buffer on a rotator for 5 min each time at 4 °C. For immunoblotting, beads were boiled in 2× Laemmli sample buffer (Bio-Rad) at 95 °C for 5 min. The eluted proteins were run on SDS-PAGE and transferred to a polyvinylidene difluoride membrane (Immobilon-P, Millipore). Proteins were incubated with GFP monoclonal antibody GF28R (Thermo Fisher Scientific) and anti-FLAG monoclonal M2 (Sigma) primary antibody and then goat anti-mouse IgG (H+L) secondary antibody conjugated with horseradish peroxidase (HRP) (Thermo Fisher Scientific). HRP was detected with the SuperSignal West Pico PLUS Chemiluminescent Substrate (Thermo Fisher Scientific).

The constructs used for co-IP were prepared by using the GreenGate method (Lampropoulos *et al*., 2013). The coding sequences of *EBO1* and *NOP58B* were amplified with the cDNA library from Arabidopsis Col-0 seedlings and cloned into the pGGC000 entry vector (Addgene ID: 48858). pGGA004 (35S promoter, Addgene ID: 48815), pGGB003 (B-Dummy, Addgene ID: 48821), pGGC-EBO1, pGGD001 (C-terminal GFP, Addgene ID: 48833), pGGE001 (rbcS terminator, Addgene ID: 48839), pGGF005 (pUBQ10::HygromycinR::tOCS, Addgene ID: 48846), and pGGZ001 (Addgene ID: 48868) were used to make the *p35S:EBO1-GFP* constructs. pGGA004 (35S promoter, Addgene ID: 48815), pGGB003 (B-Dummy, Addgene ID: 48821), pGGC-NOP58B, pGGD-3xFLAG (constructed by synthesis of 3×FLAG coding sequence and ligated into pGGD000 entry vectors, Addgene ID: 48859), pGGE001 (rbcS terminator, Addgene ID: 48839), pGGF005 (pUBQ10::HygromycinR::tOCS, Addgene ID: 48846), and pGGZ001 (Addgene ID: 48868) were used to make the *p35S:NOP58B-3xFLAG* constructs.

## Results

### Isolation and genetic analysis of *ebo1* mutants

Despite extensive functional genetic studies in Arabidopsis, approximately a third of the proteins encoded by its genome remain uncharacterized. It is likely that some of these uncharacterized genes play essential roles in fundamental cellular processes and are therefore conserved throughout the green lineage. To identify such conserved, uncharacterized, and potentially essential genes, we began by compiling a list of genes annotated with both ‘unknown molecular function’ and ‘unknown biological process’, based on TAIR 10 Gene Ontology annotations (www.arabidopsis.org). This resulted in a set of 6838 genes, of which 5279 are nuclear genes with Arabidopsis Genome Initiative (AGI) locus identifiers (Wang *et al*., 2019). To enhance the likelihood of observing mutant phenotypes via reverse genetics, we selected conserved single-copy genes from this list for further study.

To select genes conserved within the green lineage, we utilized the comparative genomics platform PLAZA (https://bioinformatics.psb.ugent.be/plaza/). We focused on single-copy genes conserved among *Arabidopsis thaliana*, the moss *Physcomitrella patens*, and the unicellular green alga *Chlamydomonas reinhardtii*. This approach identified 194 uncharacterized, evolutionarily conserved single-copy genes in Arabidopsis (for details see Wang *et al*., 2019). We further focused on genes expressed in meristems because meristematic tissues are the primary sites of active cell division and are essential for plant growth and development. Genes expressed in meristems are therefore more likely to play fundamental roles in core developmental and cellular processes. By intersecting RNA-seq and microarray datasets and restricting the analysis to meristem-expressed genes, we aimed to enrich for candidates with biologically relevant functions during early developmental stages.

**Fig. 1.**
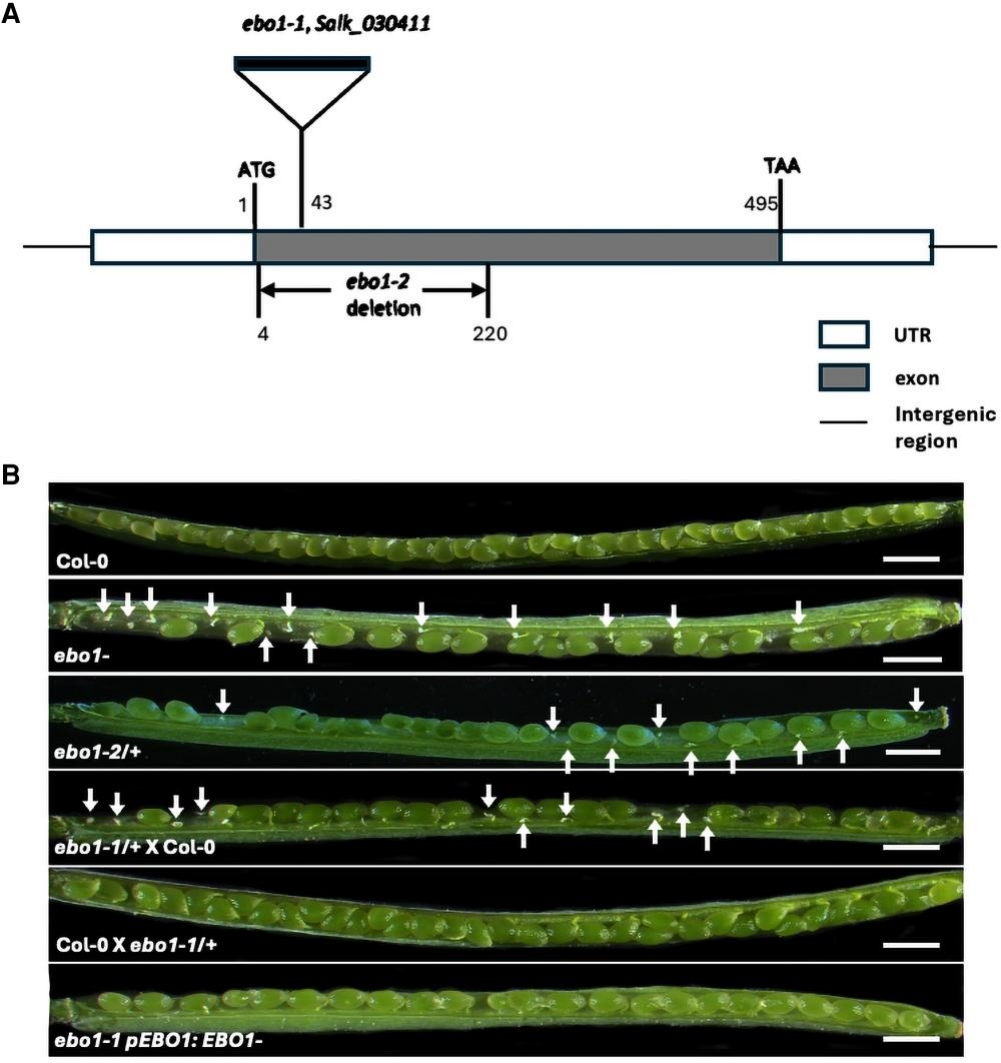
Characterization of the *ebo1* mutants of *Arabidopsis thaliana*. (A) A schematic diagram of *EBO1* gene structure, including the T-DNA insertion site in *ebo1-1* and the deletion in *ebo1-2*. The grey box indicates the exon, while the white boxes depict untranslated regions (UTRs) and black lines the intergenic region. (B) Developing seeds in siliques from Arabidopsis Col-0, *ebo1-1*/+, *ebo1-2*/+, *ebo1-1*/+×Col-0, Col-0*×ebo1-1*/+, and *pEBO1*:*EBO1-YFP* transgene complemented *ebo1-1* plants ∼7 d after pollination. White arrows indicate aborted structures. Scale bars: 1 mm.

Mutations in essential genes involved in core cellular functions often lead to gametophytic or embryonic defects, typically seen as reduced seed set. In line with this hypothesis, the reverse genetics screen outlined in Supplementary Fig. S1 identified *At1g71430* and a corresponding T-DNA mutant that displays an early seed abortion phenotype. The T-DNA insertion (SALK_030411) is located in the coding region of *At1g71430* (Fig. 1A), a single-exon gene that encodes an amino acid sequence with no significant sequence similarity to previously characterized proteins in plants. Based on publicly available gene expression data, *At1g71430* exhibits relatively higher expression in developing flowers and seeds (Supplementary Fig. S2), which is in line with the early seed abortion phenotype. To confirm the causal relationship between *At1g71430* disruption and the observed seed abortion phenotype, a second mutant allele was created using CRISPR–Cas9 gene editing. This line carries a heterozygous 217 bp (4–220) exon deletion that causes a translation frameshift in *At1g71430* resulting in a truncated protein (Fig. 1A). Heterozygous seedlings carrying the mutation, but without the CRISPR–Cas9 transgene cassette were identified and used for further analysis. The CRISPR–Cas9 mutant of *At1g71430* showed a similar seed abortion phenotype in the developing siliques as the T-DNA insertion line (Fig. 1B). Furthermore, the seed abortion was complemented by a genomic construct that corroborated the causal relationship between *At1g71430* disruption and early arrest of seed development (Fig. 1B). Thus, the gene was named *EARLY ABORTION 1* (*EBO1*), and the T-DNA insertion line and CRISPR–Cas9 generated exon deletion line are hereafter referred to as *ebo1-1* and *ebo1-2*, respectively.

To investigate the defective seed phenotype in the heterozygous *ebo1* plants in more detail, mature siliques from wild type (WT) and self-crossed as well as out-crossed *ebo1/+* plants were dissected and compared under a stereomicroscope. In addition to the normally developing seeds, some very small defective seeds or ovules were observed in the *ebo1/+* siliques. When seed development aborts very early it is difficult to distinguish aborted seeds from defective ovules. However, once the ovule is fertilized, the embryo sac begins to enlarge and the shape becomes oval, visually separating it from the smaller and triangular defective ovules. Moreover, the aborted seeds usually turn brown while the defective ovules remain white. Based on these visual cues, we scored the unfertilized ovules and aborted seeds in WT and self-crossed and out-crossed *ebo1/+* siliques. The proportions of aborted seeds and defective ovules in WT siliques were 1.4% and 0.4%, respectively (*n*=729), while the corresponding values for self-pollinated *ebo1-1*/+ siliques were 31% and 10% (*n*=583), and for the *ebo1-2*/+ 22% and 18% (*n*=552), respectively (Table 1). Moreover, when WT pollen was used to pollinate *ebo1-1*/+ pistils, 20% of seeds aborted at an early stage and 8% of ovules showed defects (*n*=737) (Table 1). This demonstrated that defects in the maternal genetic material contributed to a significant proportion of fertilization failure and seed abortion. This is in line with the genetic analysis, which revealed that the transmission rate of the *ebo1* allele through the female gametes was only 9% (Table 2). In contrast, when WT pistils were pollinated with *ebo1-1*/+ pollen, the proportions of aborted seeds and unfertilized ovules were 0.5% and 1.6%, respectively (*n*=753) (Table 1), which did not significantly differ from the results observed in self-pollinated WT siliques. However, the genetic analysis revealed that *ebo1-1* allelic transmission through the male gametes reached only 31% (Table 2), indicating that pollen function was also affected.

**Table 1.** Seed development phenotypes in *ebo1-1/+* and *ebo1-1* complemented with pEBO1:EBO1-YFP construct

Crosses (female×male)	Normal (%)	Aborted (%)	Unfertilized (%)	*n*
Col-0 self	98.2	1.4	0.4	729
*ebo1-1*/+ self	58.7	31.4	9.9	583
*ebo1-2*/+ self	60,7	21,6	17,8.	552
Col-0*×ebo1-1*/+	97.9	0.5	1.6	753
*ebo1-1*/+×Col-0	71.6	19.9	8.4	737
*ebo1-1* complemented	97.9	1.2	0.9	563

For the self-crossed Col-0, 10 siliques about 10 days after pollination (DAP) were taken randomly from three plants. For self-crossed *ebo1-1*/+, 11 siliques about 10 DAP were taken randomly from two plants. For self-crossed *ebo1-2*/+, 12 siliques about 10 DAP were taken randomly from two plants that had been determined not to contain the transgenic CRISPR cassette. For out-crossed Col-0 and *ebo1-1*/+, 11 and 12 siliques about 10 DAP were taken from three plants. For the complemented *ebo1-1*, 10 siliques about 10 DAP were taken from one plant. *n*, number of seeds and ovules observed.

**Table 2.** Genetic analysis of *ebo1-1/+* mutant

Crosses (female×male)	With insertion	Without insertion	Ratio	Expected ratio	TE (%)	χ^2^
*ebo1-1*/+ self	54	88	0.61: 1	3: 1	NA	68.92^a^
Col-0*×ebo1-1*/+	31	100	0.31: 1	1: 1	31	22.96^a^
*ebo1-1*/+×Col-0	12	138	0.09: 1	1: 1	9	53.84^a^

Transmission efficiency (TE)=(number of progeny with insertion/number of progeny without insertion)×100. χ^2^, chi-square test statistic calculated for the expected ratio. NA, not applicable.

^*a*^Results differ significantly from the expected ratio (*P*<0.01, 1 degree of freedom).

### *Ebo1* mutation affects sperm cell migration and pollen tube growth *in vivo*

The *ebo1/+* pollen was analysed in more detail to elucidate the pollen defects. Alexander staining of pollen grains showed that *ebo1/+* and WT pollen exhibited similar morphology and viability (Supplementary Fig. S3). An *in vitro* germination assay showed that 72% (*n*=812) of *ebo1-1*/+ and 69% (*n*=756) of *ebo1-2*/+ pollen germinated, which was a slightly higher than the 61% (*n*=830) observed for WT pollen. Thus, it can be concluded that the *ebo1* mutation does not affect pollen viability or germination rate *in vitro*. However, we observed that in some of the *in vitro* germinated *ebo1/+* pollen the nuclei entered into the pollen tube much later than in WT. This delay was particularly obvious for the sperm cells, which were observed in the pollen grain still after ∼10 h of pollen tube growth when they were up to 400 μm long (Supplementary Fig. S4). In pollen and pollen tubes the two sperm cells are held together by a shared extracellular matrix; one of the two sperm cells extends a thin cytoplasmic projection into the invaginated vegetative nucleus to form a complex termed male germ unit (MGU) (Russel and Cass, 1981; McCue *et al*., 2011). This structure is thought to hold the three nuclei close to each other in the growing pollen tube. The MGU nuclei have been shown to enter the pollen tube when it reaches a length of ∼5 μm and the two sperm nuclei closely follow the vegetative nucleus (Ge *et al.*, 2011). In WT the MGU follows the growing pollen tube tip (Supplementary Fig. S4A). However, in *ebo1/+* pollen tubes the sperm cells were in several cases not observed close to the tip (Supplementary Fig. S4). Quantification of the distance between the sperm cells and the pollen tube tip in WT and *ebo1-1*/+ confirmed that the *ebo1-1*/+ sperm cells were further from the tip compared with WT. In WT the average distance was 90 μm while in *ebo1-1*/+ it was 139 μm (Supplementary Fig. S4). In two-thirds (41/62) of the WT pollen tubes, the distance of sperm nuclei and pollen tip was below 100 μm, while in the *ebo1-1*/+ pollen tubes only one-third (22/62) was in this range. A sperm nuclei to pollen tube tip distance longer than 150 μm was more prevalent in *ebo1-1*/+ (26/62) compared with WT (10/62), and also in the range longer than 200 μm (12/62 in *ebo1-1*/+ *vs* 4/62 in WT) (Supplementary Fig. S4). Thus, these results showed that nucleus movement during pollen germination was affected by the *ebo1* mutation.

**Fig. 2.**
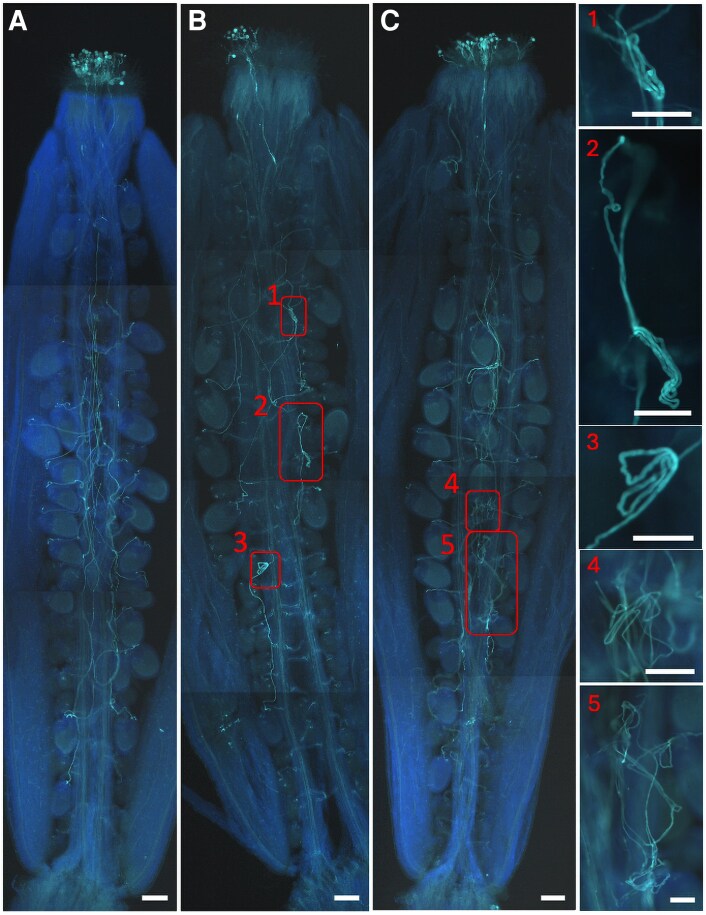
Pollen tube growth in pistils. (A–C) Wild type (WT) pistils pollinated with pollen from WT (A), *ebo1-1*/+ (B), and *ebo1-2*/+ (C), and stained with aniline blue after 36 h to visualize the growth of pollen tubes. The areas marked with red squares are magnified on the right with the same numbers. The images are representative of three replicate experiments. Scale bars in (A–C): 100 μm; scale bars in (1–5): 50 μm.

An *in vivo* germination assay was performed to assess the *ebo1*/+ pollen performance in the pistils. A small amount of pollen from WT or *ebo1*/+ was applied to the WT pistils, and after 36 h the pistils were collected and stained with aniline blue to visualize the pollen tubes. Almost all the pollen tubes could grow in the pistils and reach ovules (Fig. 2). However, in the *ebo1*/+ pollinated pistils several pollen tubes were observed to grow back and forth in a pattern that was not observed in the pistils pollinated with WT pollen (Fig. 2). This observation provides a plausible explanation for the lower-than-expected paternal transmission rate of *ebo1*, since the *ebo1* pollen tubes are more likely to be outcompeted by WT pollen tubes *in vivo*.

### The *ebo1* mutation affects ovule and seed development

**Fig. 3.**
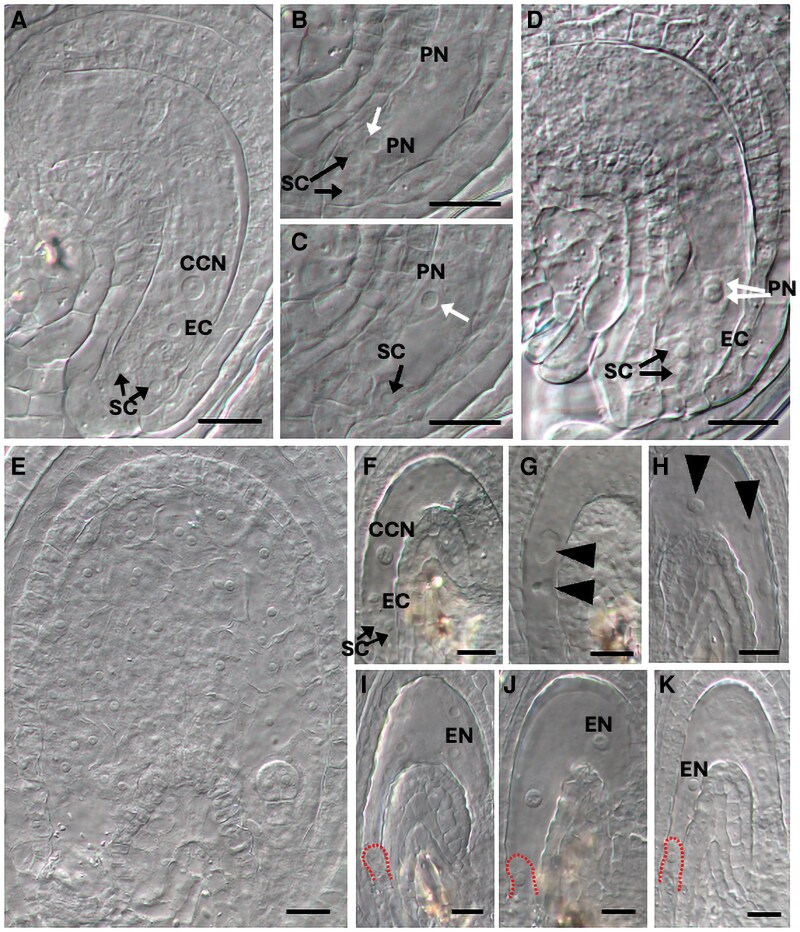
Ovules and developing seeds from Arabidopsis Col-0 and *ebo1-1/+* plants. (A) A Col-0 ovule with a central cell nucleus (CCN), one egg cell (EC), and two synergid cells (SC). (B) Ovule defects in *ebo1-1/+* samples. One ovule with three nuclei at the chalazal end (black arrows), and one of which should be a polar nucleus (PN, white arrow). (C) Different focal plane of the ovule in (B) showing a fourth nucleus at the chalazal end (black arrow) and the same polar nucleus (white arrow) as in (B). This indicates that the two polar nuclei did not fuse. (D) *ebo1-1/+* samples showing two unfused polar nuclei in close proximity. (E) Normally developing seed in an *ebo1-1/+* silique 2 d after pollination at the two- or four-cell embryo stage. (F) Unfertilized ovule in the same *ebo1-1/+* silique as in (E). (G, H) Fertilized central cell nucleus divided into two nuclei with different sizes and irregular shapes (black triangles) in *ebo1-1/+* silique as in (E). (I) Developing seed in *ebo1-1/+* silique as in (E) with several endosperm nuclei and one non-elongated zygote. (J) Developing seed in *ebo1-1/+* silique as in (E) with two endosperm nuclei and an elongated zygote. (K) Developing seed in *ebo1-1/+* silique as in (E) with several endosperm nuclei and one elongated zygote. Red dotted lines outline the zygotes. EN, endosperm nucleus. Scale bars: 50 μm.

To further analyse the ovule and seed development defects, unpollinated ovules and immature seeds from WT and *ebo1/+* mutant plants were observed with a differential interference contrast microscope. Arabidopsis is a self-pollinating plant in which the start of pollination precedes flower opening, and thus just prior to flower opening the ovules are fully developed and ready to be fertilized. At this stage the ovule contains a four-cell structure—one large central cell nucleus that is formed by the fusion of two polar nuclei, one egg cell, and two synergid cells (Shi and Yang, 2011). In order to observe ovule development in WT and *ebo1/+*, the stamens were removed 1 or 2 d before estimated flower opening to avoid fertilization, and then on the day of opening the pistils were collected and cleared. The ovules from pistils of WT and most *ebo1-1*/+ plants exhibited a typical four-cell structure (Fig. 3A). However, the two polar nuclei remained unfused in some *ebo1-1*/+ ovules, and were either clearly separated by the central vacuole (Fig. 3B, C) or close to each other (Fig. 3D), indicative of arrested or delayed ovule development. Similar unfused polar nuclei were also observed in *ebo1-2*/+ ovules (Supplementary Fig. S5). The polar nuclei phenotype was also observed when the mature pistils were allowed to develop one more day without pollination.

The ovule development defects observed in *ebo1* mutant pistils may result in delayed or missed fertilization. This is supported by the presence of unfertilized ovules in *ebo1-1*/+ and *ebo1-2*/+ siliques, even after 2 d of pollination (Fig. 3F; Supplementary Fig. S5). In contrast, the embryos in WT siliques and many embryos in mutant siliques were at the two- or four-cell stage at this point (Fig. 3E; Supplementary Fig. S5). Some of the fertilized ovules collected from *ebo1/+* plants showed two endosperm nuclei of irregular shape and different sizes, and without visible zygote (Fig. 3G, H). In some cases, the endosperm nuclei in fertilized ovules divided normally for several rounds while zygote development was arrested (Fig. 3I); in other cases, both zygote development and the division of endosperm nuclei were arrested (Fig. 3J, K; Supplementary Fig. S5). These slowly developing seeds would likely experience developmental arrest, as in the more mature *ebo1/+* siliques we only observed early seed abortion or normal seeds (Fig. 1B).

### EBO1–YFP localizes to the nucleus

**Fig. 4.**
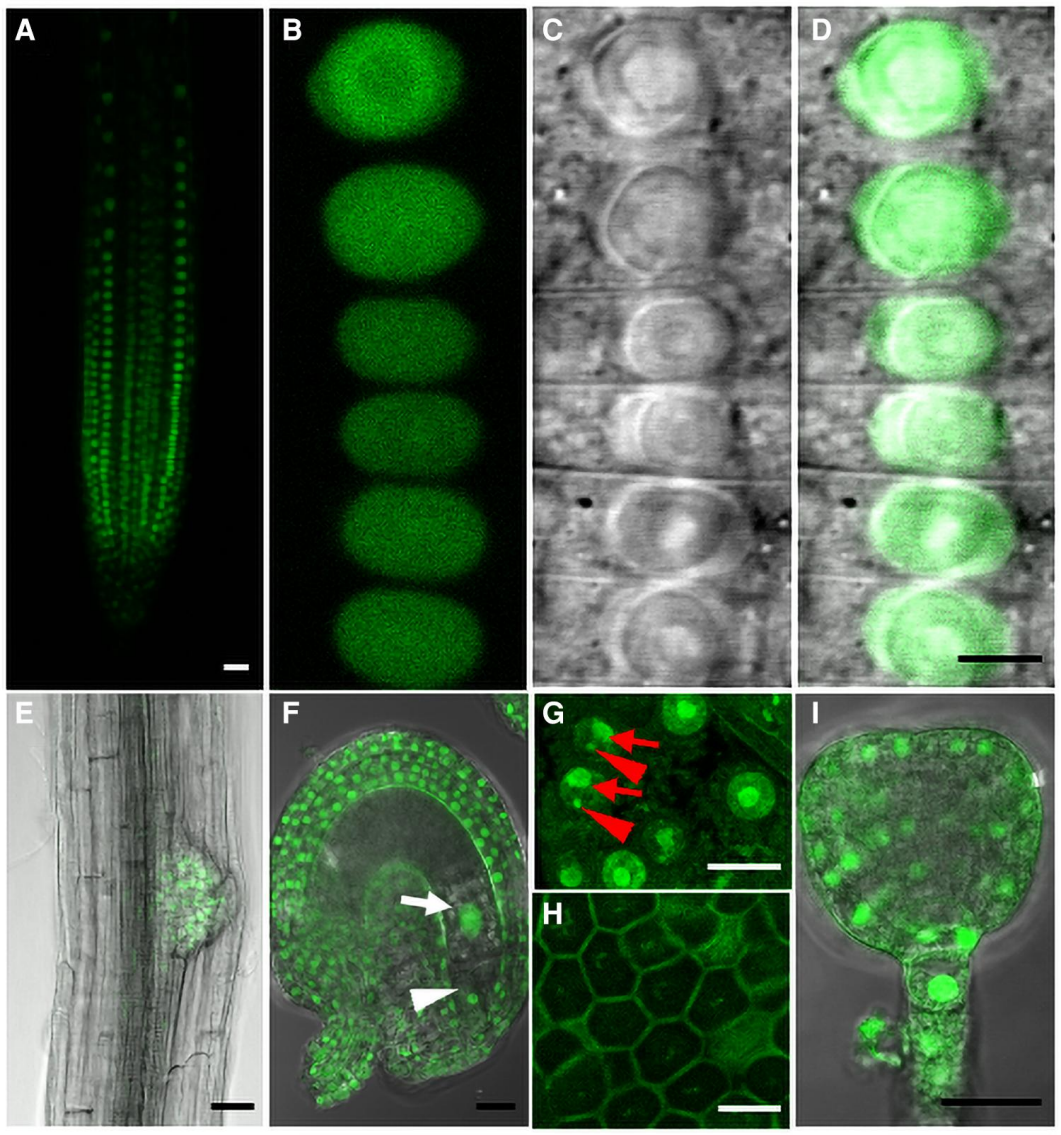
Confocal laser scanning microscope images of the EBO1–yellow fluorescent protein (YFP) signal from Arabidopsis tissues undergoing cell proliferation. (A) Root tip of a seedling expressing *pEBO1:EBO1-YFP*. (B) Close-up of the EBO1–YFP signal from root nuclei. (C) Bright-field image of the root nuclei. (D) Overlaid image of (B) and (C). (E) EBO1–YFP signal in lateral root primordium. (F) EBO1–YFP signal in an unfertilized ovule, showing the signal in central cell nucleus and egg cell. (G) EBO1–YFP signal in bicellular pollen, showing the signal in vegetative nuclei and sperm nuclei. (H) EBO1–YFP signal in tricellular pollen before release from anther, showing the signal in two sperm nuclei. (I) EBO1–YFP signal in a developing embryo. The white arrow indicates the central cell nucleus, and the white triangle indicates the egg cell; the red arrows indicate the vegetative nuclei, and the red triangles indicate the sperm nuclei. Scale bars: 20 μm.

To investigate the subcellular localization of EBO1, a YFP coding sequence was fused to the C-terminus of EBO1 and expressed under the control of the native promoter (*pEBO1::EBO1-YFP*). Expression of this construct complemented the *ebo1* seed abortion phenotype (Fig. 1B; Table 1), and thus established that the EBO1–YFP fusion protein is functional. In the root tip cells of the complemented *ebo1* plants, the EBO1–YFP signal localized to the nucleus, which suggests a function in nuclear processes (Fig. 4A–C). The nuclear EBO1–YFP signal was also prominent in the lateral root primordium, ovules, and embryos (Fig. 4G–J). In unfertilized ovules, the EBO1–YFP signal was clearly observable in both the central cell nucleus and the egg cell nucleus (Fig. 4H). Thus, the EBO1–YFP fluorescence signal suggests that EBO1 is present in tissues undergoing cell proliferation. The observed signal was also consistent with the *ebo1* phenotypes associated with delayed or failed fertilization and defects in the division of the fertilized central cell nucleus and egg cell.

### EBO1-associated proteins point to nuclear function

**Fig. 5.**
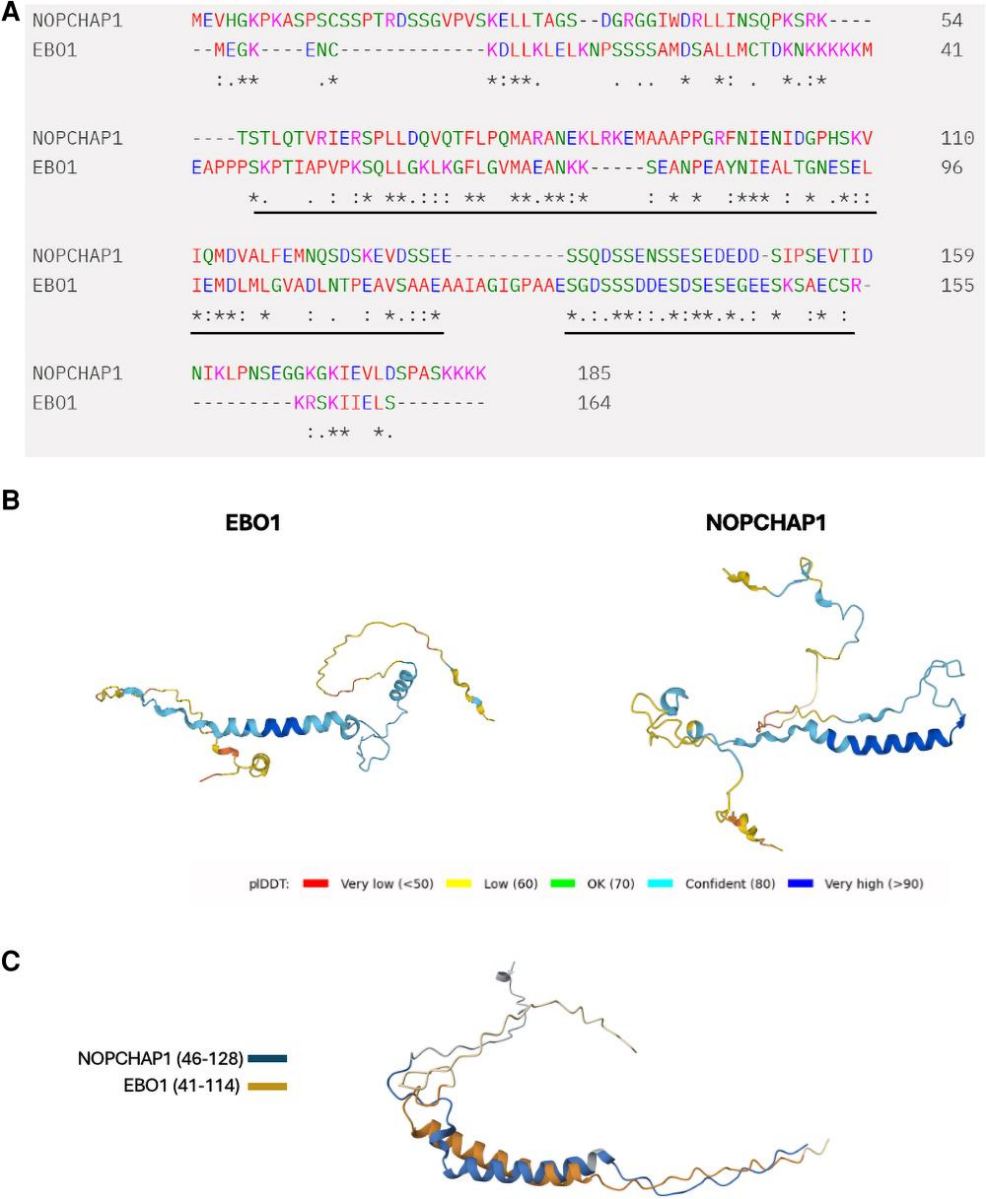
Predicted structures of EBO1 and NOPCHAP1. (A) Pairwise structural alignment of EBO1 and NOPCHAP1; highly similar parts are labelled by black lines. (B) AlphaFold predicted structures of the Arabidopsis EBO1 and human NOPCHAP1. Color coding marks per-residue model confidence score (pIDDT). (C) The predicted similar α-helix in the middle domain of NOPCHAP1 and EBO1.

*EBO1* encodes a small protein of 164 amino acids and a predicted size of ∼17 kDa. It contains a domain of unknown function, DUF4598, which has been found in many species, including humans. In humans, the DUF4598 domain is found in NOP protein chaperone 1 (NOPCHAP1) (Supplementary Fig. S6). However, EBO1 and NOPCHAP1 only show 29% identity and 50% similarity over 83 amino acids in the middle of the proteins (Fig. 5A). The 3D structure of EBO1 predicted using AlphaFold depicts an α-helix in the middle domain (high prediction confidence), while the N- and C-terminal regions show disordered structures (low prediction confidence) (Fig. 5B). The 3D structure predicted for NOPCHAP1 also shows a similar middle α-helix and disordered N- and C-terminal ends (Fig. 5B). A pairwise structural alignment between EBO1 and NOPCHAP1 reveals some degree of similarity, especially in middle parts of the proteins (Fig. 5C), which is consistent with the comparison of sequence similarity.

**Fig. 6.**
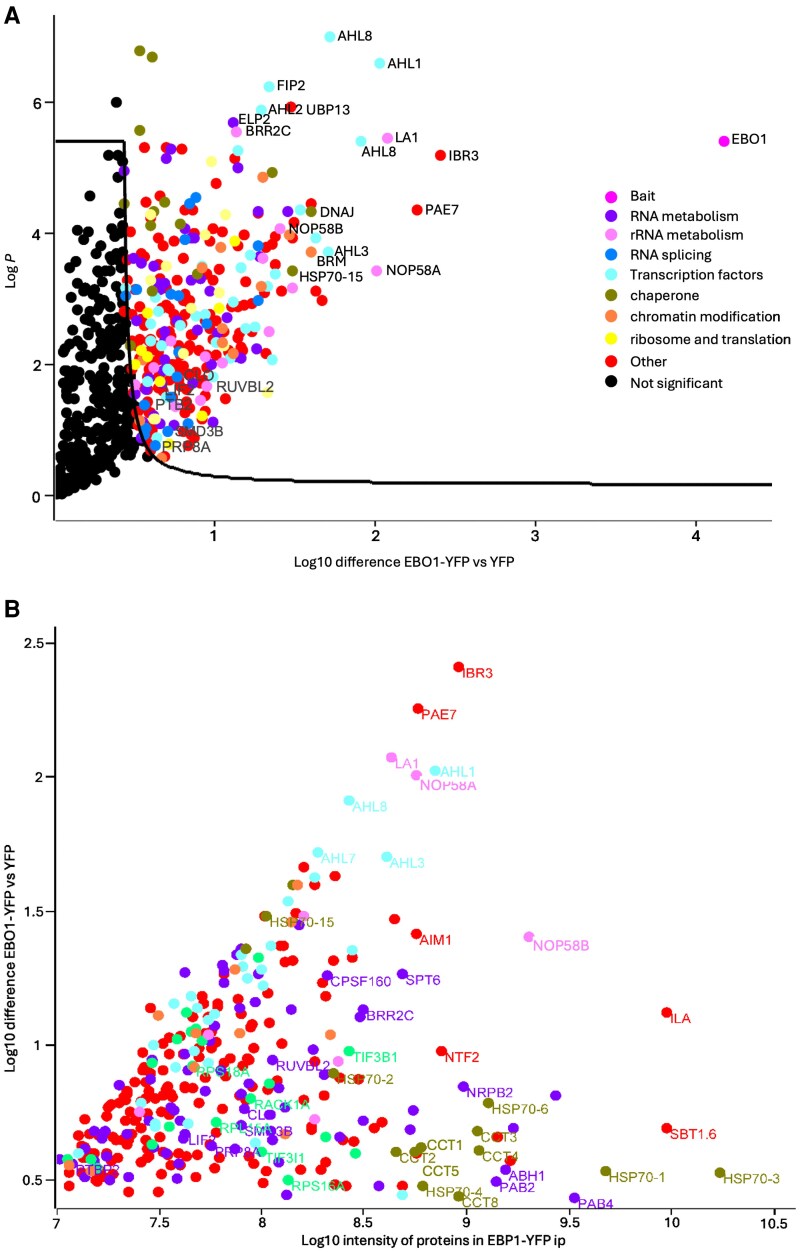
Mass spectrometry identification and quantification of EBO1–yellow fluorescent protein (YFP) co-immunoprecipitated proteins. (A) A volcano plot of the −log10 *P*-value (*y*-axis) of protein level differences and the enrichment in EBO1–YFP samples versus YFP control samples (*x*-axis). The black line denotes the set threshold for statistically significant difference determined by Student’s *t*-test moderated by the Benjamini–Hochberg method. The false discovery rate was set to 0.05 and S0 was set to 1. (B) Scatter plot of the differences in protein intensity in EBO1–YFP vs YFP samples, as log10 intensity difference (*y*-axis) and the log10 intensity of proteins identified in the EBO1–YFP co-immunoprecipitation analyses (*x*-axis). Each dot represents a protein. Different colors are used to classify the annotated functions of distinct proteins. The full list of proteins is available in Supplementary Dataset S1.

To shed further light on the function of EBO1, we isolated the EBO1–YFP fusion protein and associated proteins from the callus of *pEBO1::EBO1-YFP* transgenic plants using GFP-Trap beads. The isolated proteins were identified by mass spectrometry and quantified using a label-free quantification method (Yu *et al*., 2021). Co-IP using callus from free YFP-expressing plants served as a control to filter non-specific interactors from the dataset. The co-IP experiment identified a total of 334 proteins with the EBO1 as the most enriched protein as expected (Fig. 6; Supplementary Dataset S1). Protein function analysis showed that the EBO1-associated proteins are enriched in RNA metabolism, including transcription, RNA modification, and RNA processing, along with chromatin modification, protein translation, and folding. A recent study focusing on human NOPCHAP1 also identified putative interacting proteins using co-IP and mass spectrometry (Abel *et al*., 2021). A comparison of the proteins found to interact with EBO1 and NOPCHAP1 based on co-IP identified potential common interactors (Supplementary Table S2). These proteins include RuvB-like AAA-ATPase (RUVBL), Nucleolar Protein 58 (NOP58), components of box C/D small nucleolar ribonucleoprotein (snoRNP), U5 small nuclear ribonucleoprotein (snRNP), heterogeneous nuclear ribonucleoprotein (hnRNP), heat shock protein of 70 kDa (HSP70), T-complex protein-1 ring complex/chaperonin containing TCP1 (TRiC/CCT) chaperones, and ribosome- and translation-related proteins. These shared interactors support the hypothesis that EBO1 and NOPCHAP1 share similar molecular functions.

**Fig. 7.**
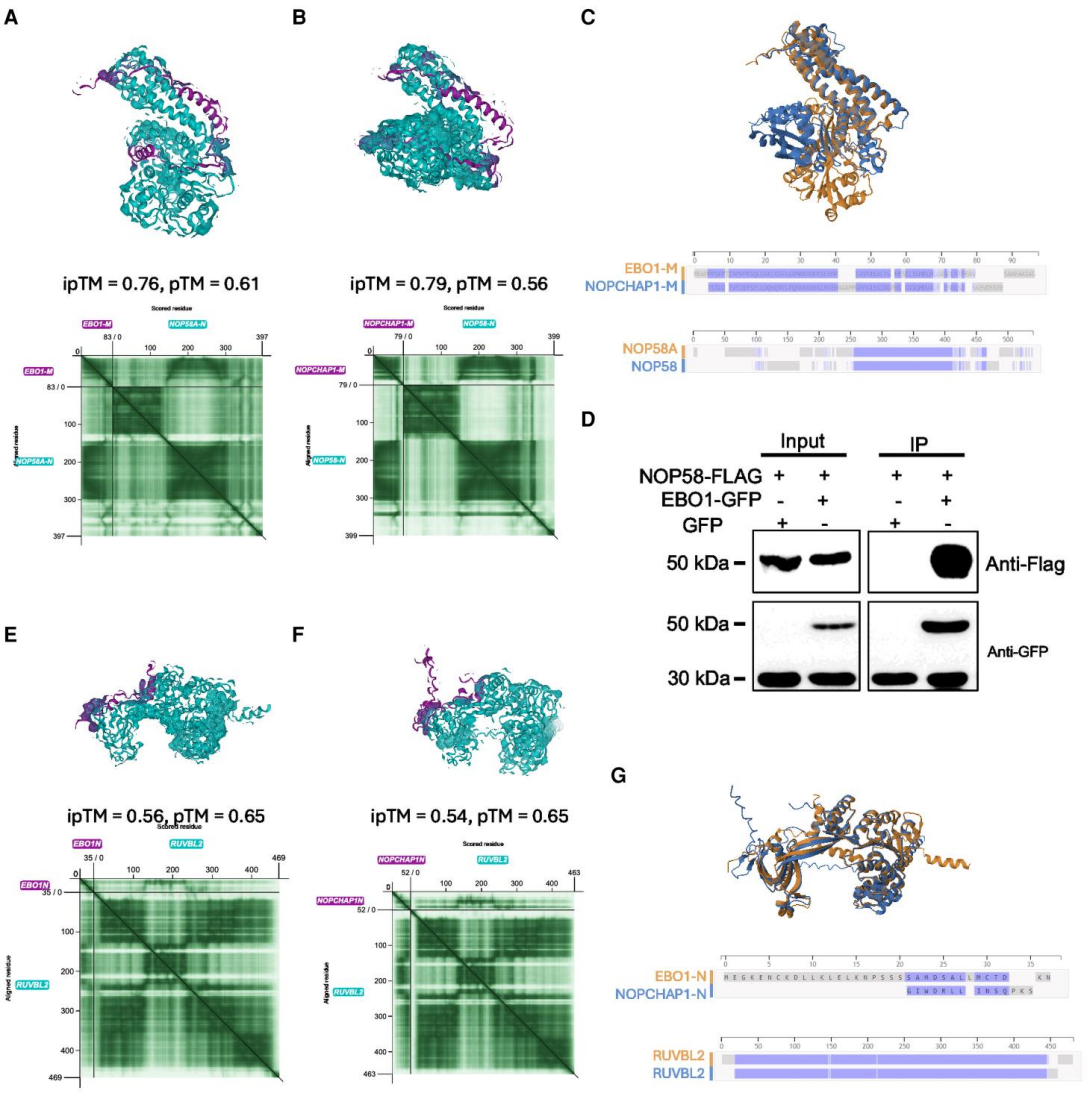
Structural models of the complexes formed by EBO1 and NOP58A or NOPCHAP1 and NOP58. (A, B) AlphaFold-predicted structure of the dimer formed by EBO1 middle part (EBO1-M) and NOP58A N-terminal part (NOP58A-N) (A) or the dimer formed by NOPCHAP1 middle part (NOPCHAP1-M) and NOP58 N-terminal part (NOP58-N) (B). (C) Pairwise structure alignment of the 3D structures of the two dimers, EBO1-M–NOP58A-N and NOPCHAP1-M–NOP58-N. The alignments of EBO1-M/NOPCHAP1-M and NOP58A-N/NOP58-N sequences are shown below. (D) Western blot analysis of co-immunoprecipitation to assess NOP58–FLAG and EBO1–green fluorescent protein (GFP) interaction after *Agrobacterium-*mediated transient expression in tobacco (*Nicotiana benthamiana*) leaves. Free GFP was used as a control. (E, F) AlphaFold predicted structure of the dimer formed by EBO1 N-terminal part (EBO1N) and RUVBL2 from Arabidopsis (E) or the dimer formed by NOPCHAP1 N-terminal part (NOPCHAP1N) and RUVBL2 from human (F). (G) Pairwise structure alignment of the 3D structures of the two dimers, EBO1N–RUVBL2 and NOPCHAP1N–RUVBL2. The alignments of EBO1-N/NOPCHAP1-N and RUVBL2/RUVBL2 sequences are shown below. The interface-predicted template modeling (ipTM) scores, and the predicted template modeling (pTM) scores are also shown. The predicted aligned error (PAE) plots provided for each predicted structure demonstrate the high confidence (dark green) and low confidence (pale green) regions for the predicted structure. The color coding indicates the protein identity as shown in the PAE plots.

The interaction between EBO1, NOP58A, NOP58B, and RUVBL2 in Arabidopsis is also supported by the overlapping expression pattern of the corresponding genes in various tissues including seeds, embryos, and root tip meristem cells (Supplementary Fig. S7). To further investigate the interaction between EBO1 and the putative interactors, we used the protein structure prediction tool AlphaFold to model the possible protein complexes. For each prediction, the interface predicted template modeling (ipTM) score, and the predicted template modeling (pTM) score were used to assess the quality of the predicted structures. Predicted aligned error (PAE) plots depict the confidence in relative positions of the predicted structures. The modeling results predicted that EBO1 and NOP58A form a dimer with relatively high confidence scores (ipTM=0.53, pTM=0.56; Supplementary Fig. S8). Furthermore, the human NOPCHAP1 was also predicted to interact with the human NOP58 (ipTM=0.48, pTM=0.46; Supplementary Fig. S8). Since the prediction quality of the N-terminal and C-terminal parts of EBO1/NOPCHAP1 and the C-terminal parts of NOP58A/NOP58 is low (the pLDDT scores are low; Supplementary Fig. S8A, B) and these parts are not involved in the interactions, they were removed prior to repeating the complex modeling. This led to higher confidence scores of the predicted 3D structures for both EBO1 Middle-NOP58A N-terminus (ipTM=0.76, pTM=0.61; Fig. 7A) and NOPCHAP1 Middle-NOP58 N-terminus (ipTM=0.79, pTM=0.56; Fig. 7B). Furthermore, a comparison of the dimer structures revealed a similar 3D structure in both cases (Fig. 7C). To verify that EBO1 and NOP58B can interact *in vivo*, we transiently expressed EBO1–GFP and NOP58B–3×FLAG in tobacco (*Nicotiana benthamiana*) leaves. Free GFP was co-expressed with NOP58B–3×FLAG as a control. Co-IP with GFP-Trap beads followed by western-blot analysis showed that NOP58B–3×FLAG interacted with EBO1–GFP but not with the GFP control (Fig. 7D; Supplementary Fig. S9).

In addition, we also attempted to model the complex formed by EBO1 and other candidates, but the overall scores were low for the EBO1–RUVBL2 dimer (ipTM=0.34, pTM=0.48; Supplementary Fig. S8) and the EBO1–RUVBL2–NOP58A trimer (ipTM=0.28, pTM=0.39; Supplementary Fig. S8). The scores were also low for the NOPCHAP1–RUVBL2 dimer (ipTM=0.37, pTM=0.41; Supplementary Fig. S8) and the NOPCHAP1–RUVBL2–NOP58 trimer (ipTM=0.23, pTM=0.37; Supplementary Fig. S8). But the results also suggested that the N-terminal parts of EBO1 and NOPCHAP1 may interact with RUVBL2, which was supported by the high confidence score in the PAE plots (Supplementary Fig. S8). The N-terminal parts of EBO1 (EBO1N) and NOPCHAP1 (NOPCHAP1N) were used to predict 3D structure together with RUVBL2 from Arabidopsis and human, respectively. The results showed higher scores for EBO1N–RUVBL2 (ipTM=0.56, pTM=0.65; Fig. 7E) and NOPCHAP1N–RUVBL2 (ipTM=0.54, pTM=0.65; Fig. 7F). The interfaces of EBO1N–RUVBL2 and NOPCHAP1N–RUVBL2 were also similar (Fig. 7G). Thus, taken together the interaction, gene expression, co-IP, and modeling results show that EBO1 can interact with NOP58A and possibly also with RUVBL2 in plants.

## Discussion

Our results demonstrate the potential of using reverse genetic screens with a sufficiently narrow focus to explore the uncharacterized genome space in Arabidopsis. *EBO1* is an evolutionarily conserved gene with an unknown function. The homozygous *ebo1* knockout mutant line is lethal, and the divisions of endosperm nuclei and embryo proceed slowly and arrest at early stages. The low transmission rates of *ebo1* mutation through both male and female gametes showed that EBO1 is also required for the development and function of gametophytes. Unfused polar nuclei, which can cause delayed or failed fertilization, were often observed in ovules of *ebo1/+* mutants. We also observed a maternal effect on the abortion of seed development at a very early stage (Fig. 1B). Furthermore, only 31% of *ebo1* alleles were transmitted to the next generation through the male gametophyte (Table 2). We hypothesize that this low transmission rate is linked to the meandering *in vivo* growth of *ebo1* mutant pollen tubes in pistils (Fig. 2). It is also possible that the late entry of the nuclei into pollen tube also contributes to paternal transmission defect.

*EBO1* is ubiquitously expressed in plant tissues and organs, with predominant expression observed in dividing cells; this is indicative of roles that extend beyond reproductive tissues. The *EBO1-YFP* transgene was able to rescue the *ebo1* phenotype, and confocal microscopy images demonstrated that EBO1–YFP mainly localizes to the nucleus, including both the nucleolus and nucleoplasm. EBO1–YFP co-IP analyses, followed by mass spectrometry, identified 334 putative EBO1 interactors, most of which were predicted to localize to the nucleus. These interactors provided important clues for understanding the function of EBO1. Most of the interactors (111 out of 334) are involved in RNA metabolism, more specifically, RNA synthesis, processing, modification, and transport, while the results also suggest that EBO1 interacts with protein chaperones, such as HSP70 proteins and TRiC/CCT complex subunits. In addition, the co-IP results included proteins involved in chromatin modification, for example chromatin remodeling and acetylation.

The human NOPCHAP1 is currently the only homolog of EBO1 that has been functionally characterized in some detail. Thus, a comparison of the results from co-IP analyses focusing on EBO1 and the human NOPCHAP1, when combined with predictions of structural similarity, could point to conserved functions across kingdoms. The predicted structures of EBO1–NOP58A from Arabidopsis and NOPCHAP1–NOP58 from human are similar. Furthermore, the contact sites between EBO1 and NOP58A are also similar to the contact sites between NOPCHAP1 and NOP58 (Fig. 7). These results suggested that the complex is conserved between plants and animals. The human NOP58 is a core component of box C/D small nucleolar ribonucleoproteins (snoRNPs) required for ribosomal RNA modification and processing (Yu *et al.* 2018). The function of NOP58A and NOP58B in plants is unclear but based on the homology with the human NOP58, they may also play a role in ribosomal RNA modification and processing.

Another shared interactor between the plant protein EBO1 and the human protein NOPCHAP1 was RuvB-like helicase (RUVBL), which is an AAA-ATPase and ATP-dependent DNA helicase. The predicted structure also indicated the N-terminus of EBO1 may interact with RUVBL2 (Supplementary Fig. S8C, D). Human RUVBL1 and RUVBL2 play roles in many cellular activities; for instance, they are components of the INOsitol auxotrophy 80 (INO80) and SWI2/SNF2-Related 1 (SWR1) chromatin remodeling complexes (Jin *et al*., 2005; Chen *et al*., 2011; Obri *et al*., 2014; Willhoft and Wigley, 2020), as well as the NuA4 histone acetyltransferase complex (Doyon *et al*., 2004). RUVBLs are also important regulators of the Fanconi anemia core complex, which is involved in DNA damage repair (Rajendra *et al*., 2014). Moreover, RUVBLs play important roles in the assembly of protein complexes, for example telomerase holoenzyme, mammalian target of rapamycin (mTOR) complex, box C/D small nucleolar ribonucleoprotein (box C/D snoRNP), and U5 small nuclear ribonucleoprotein (U5 snRNP) (Venteicher *et al*., 2008; Kim *et al*., 2013; Cloutier *et al*., 2017; Shin *et al*., 2020; Abel *et al*., 2021). In Arabidopsis, *ruvbl1* and *ruvbl2* mutant lines show defects in both female and male gametophytes (Dvořák Tomaštíková *et al*., 2023) that are reminiscent of the *ebo1* phenotype (Fig. 3; Supplementary Fig. S5). Interestingly, the EBO1 co-IP experiments also listed RUVBL-related proteins, for example subunits of SWI/SNF chromatin remodeling complexes like BRM, SYD, SWI3D, and SWP73B (Guo *et al*., 2022), as well as components of box C/D snoRNP (NOP58A and NOP58B) and U5 snRNP (BRR2C, CLO, PRP8A, and SMD3B). Components of box C/D snoRNP and U5 snRNP were also identified in the NOPCHAP1 co-IP experiments, which suggests shared functional roles between EBO1, RUVBLs, and specific RNPs across the plant and animal kingdoms. Taken together, these data suggest that EBO1 may function as a RUVBL cofactor. In the human study, NOPCHAP1 was proposed to be a PAQosome cofactor that loads NOP58 for proper box C/D snoRNP assembly. The PAQosome has been characterized as a chaperone for the assembly of multiprotein complexes in mammalian cells (Houry *et al*., 2018; Abel *et al*., 2021). However, no homologs of PAQosome core subunits were identified in the EBO1 co-IP dataset. The putative EBO1 associated proteins also included 13 AT-HOOK MOTIF NUCLEAR LOCALIZED (AHL) proteins, which are specific to plants and associated with a wide range of plant growth and development processes (Zhang *et al*., 2022), including embryo development in Arabidopsis (Karami *et al*., 2021). The EBO1-associated candidates also included AtLa1, a 3′-UUU-OH RNA-binding protein involved in RNA processing. Arabidopsis *atla1* null mutants are embryonic-lethal (Fleurdepine *et al*., 2007), which could hint at a possible link to EBO1 function.

In summary, our results establish an important role for *EBO1* in cell proliferation in general, and gamete and seed development in particular. Co-IP and 3-D modeling identified likely EBO1 interactors, which serve as a basis for further research into this previously uncharacterized vital process in plant cells.

## Supplementary Material

erag142_Supplementary_Data

## Data Availability

The datasets generated during and/or analysed during the current study are included as supplementary material and/or available from the corresponding author on reasonable request.

## Supplementary data

The following supplementary data are available at *JXB* online.

Fig. S1. Outline of the genetic screen.

Fig. S2. Expression pattern of *EBO1.*

Fig. S3. Alexander staining of pollen and *in vitro* pollen germination.

Fig. S4. Distance between sperm cells and pollen tube (PT) tips.

Fig. S5. Ovules and developing seeds from *ebo1-2/+* plants.

Fig. S6. Phylogenetic tree of DUF4598 containing proteins.

Fig. S7. Expression pattern of *EBO1*, *NOP58A*, *NOP58B*, and *RUVBL2*, and expression profiles in root of *EBO1* and the genes coding for the candidate interactors

Fig. S8. Structural models of the complexes formed by EBO1.

Fig. S9. Complete immunoblots of the EBO1–GFP and Nop58–FLAG co-immunoprecipitation experiments.

Table S1. List of primers used in the study.

Table S2. Common interactors between Arabidopsis EBO1 and human NOPCHAP1.

Dataset S1. EBO1–GFP co-immunoprecipitation enriched proteins.
