# Supplementary material for: EARLY ABORTION 1 is an evolutionarily conserved gene required for plant reproduction
Source: J Exp Bot. 2026 Mar 23;77(14):4493–507. doi: 10.1093/jxb/erag142 (PMC13415960; doi:10.1093/jxb/erag142)
Supplement: erag142_Supplementary_Data [file erag142_supplementary_data.zip › jexbot317144-file001.pdf]

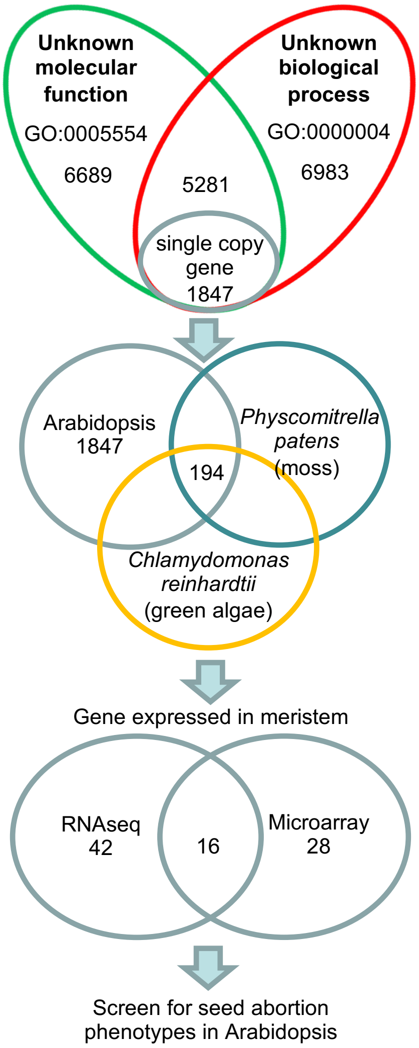

Supplementary Fig S1. Outline of the genetic screen for evolutionary conserved single genes of unknown function involved in an unknown process. Number of genes at each filtering stage is shown.

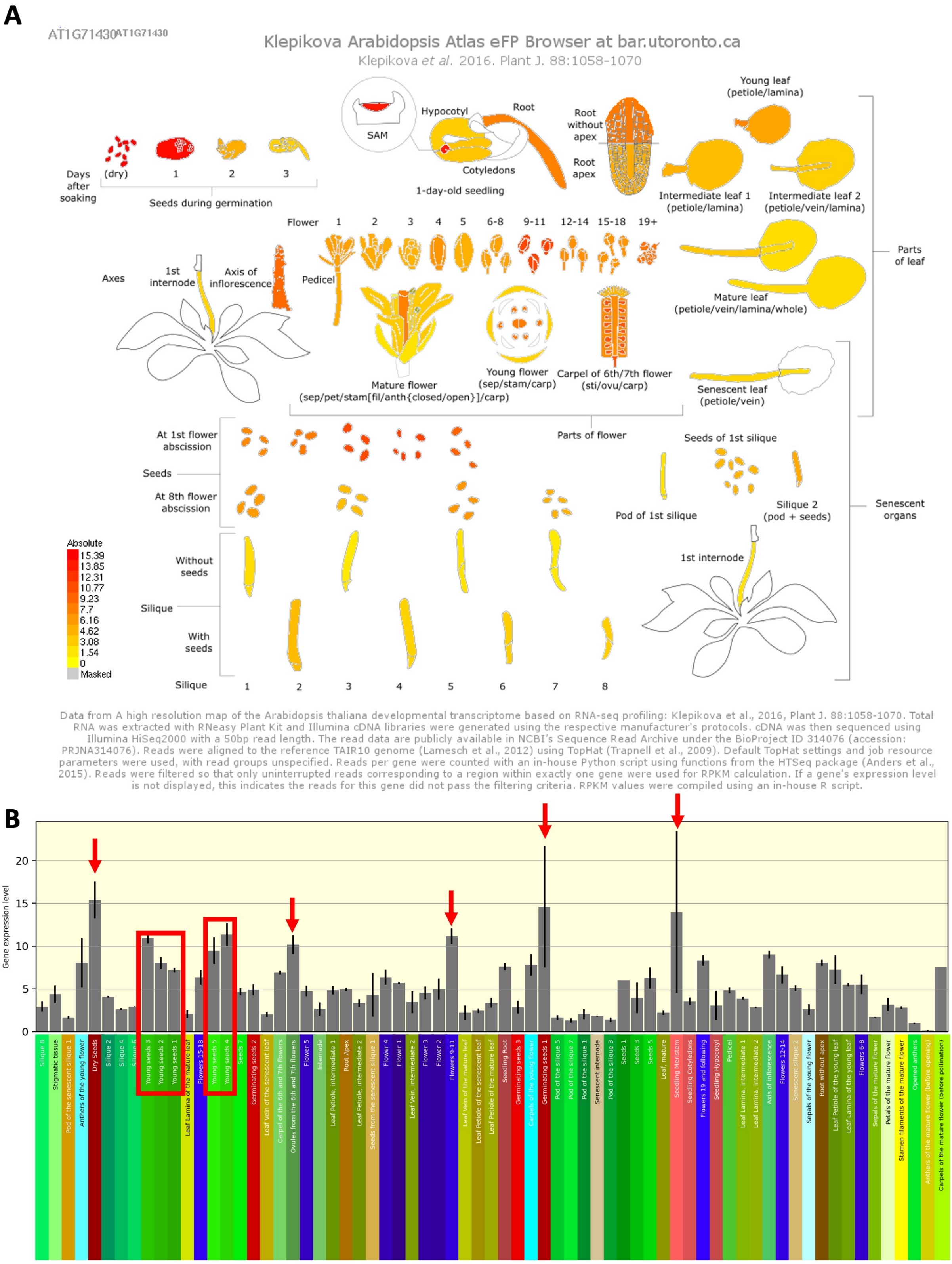

Supplementary Fig S2. Expression pattern of *EBO1* (AT1G71430) gene in different Arabidopsis tissue.

(A) Heat map of *EBO1* gene expression pattern.

(B) *EBO1* expression level in specific organs and tissues at different developmental stage, red arrows mark the ones with relatively high *EBO1* expression level, and red boxes mark the early stage of seed development with high *EBO1* expression level, which is line with the lethal phenotype of *ebo1* mutant at this stage.

Data obtained from TAIR (http://www.arabidopsis.org) (Winter *et al*. 2007) accroding to the RNA-seq data

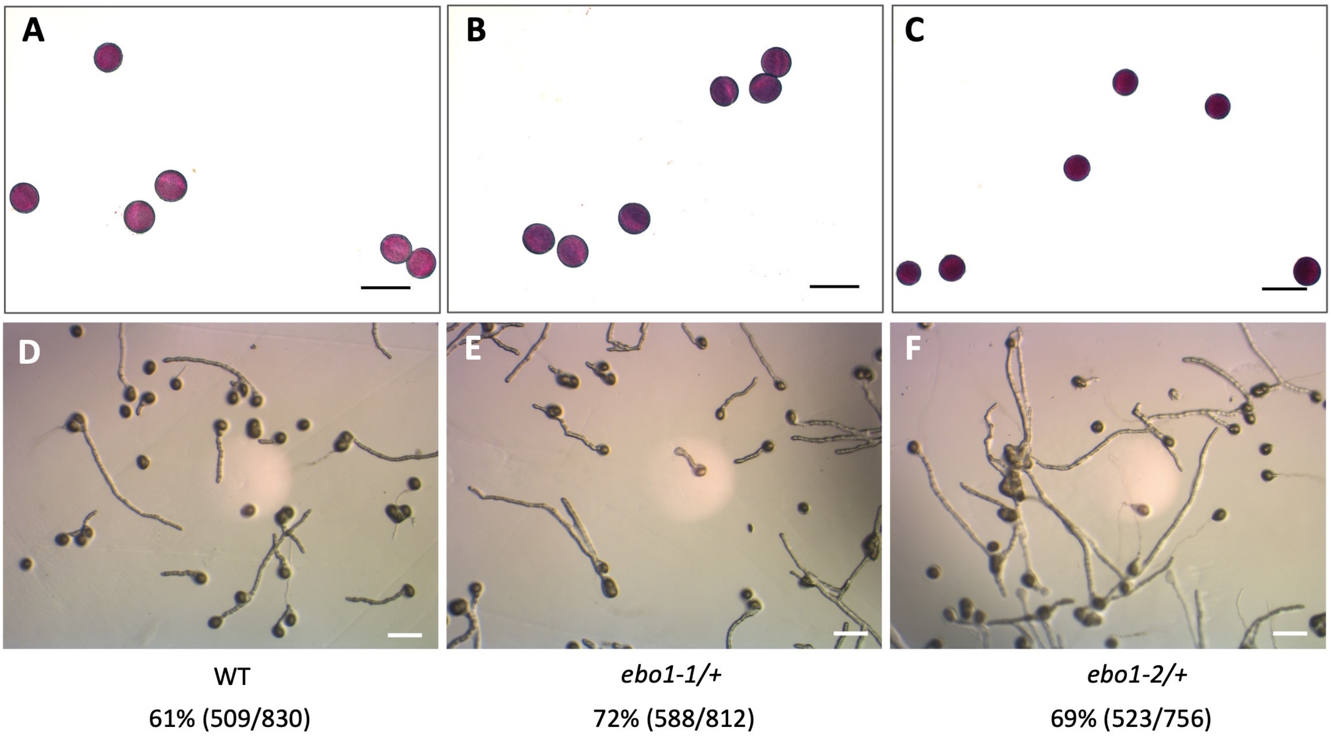

Supplementary Fig S3. Alexander staining of pollen and *in vitro* pollen germination
(A – C) Alexander staining of pollen from WT (A), *ebo1-1*/+ (B) and *ebo1-2*/+ (C), all pollens could be stained to be red, demonstrating the ebo1 mutant pollens have normal morphology and viability.
(D – F) *In vitro* pollen germination of WT (D), *ebo1-1*/+ (E) and *ebo1-2*/+ (F), pictures were taken after 5 hours.
Scale bars = 100 μm.

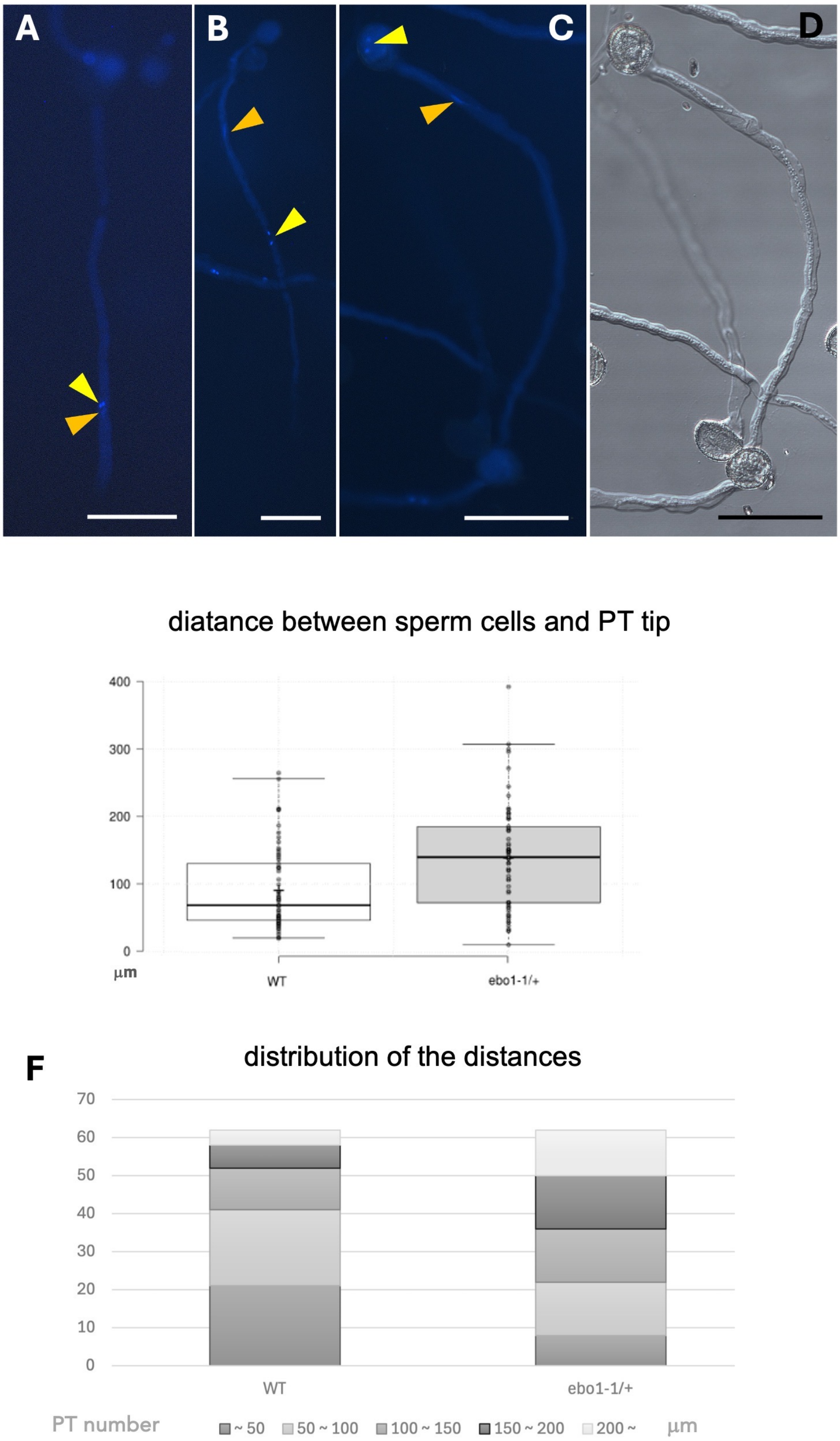

Supplementary Fig S4. Distance between sperm cells and pollen tube (PT) tips.
(**A**) Nuclei stained by DAPI in WT pollen tube, showing a typical MGU of a vegatative nuclues followed by two sperm cells closed to the pollen tube tip.**(B**) Nuclei in *ebo1-1*/+ pollen tube, showing two sperm cells are ahead of and far away from the vegatative nucleus. (**C**) Nuclei in *ebo1-1*/+ pollen tube, showing two sperm cells are still in pollen grain whereas the vegetative nucleus has entered into pollen tubes, but far away from the tip. (**D**) The same view in (C) under bright field. (**E**) Comparison of the distances from position of sperm cells to pollen tip in WT and *ebo1-1*/+, showing in many pollen tubes from *ebo1-1*/+ the sperm nuclei are further from tip than in the WT pollen tubes. 62 pollen tubes from each genotype were measured. The box plot was generated with the online tool BoxPlotR (http://shiny.chemgrid.org/boxplotr/). (**F**) Distribution of the distances in different ranges, vertical axis represents the numbers of pollen tube.
Orange triangle indicates vegatative nucleus; yellow triangle indicates sperm nucleus. Scale bars = 100 µm.

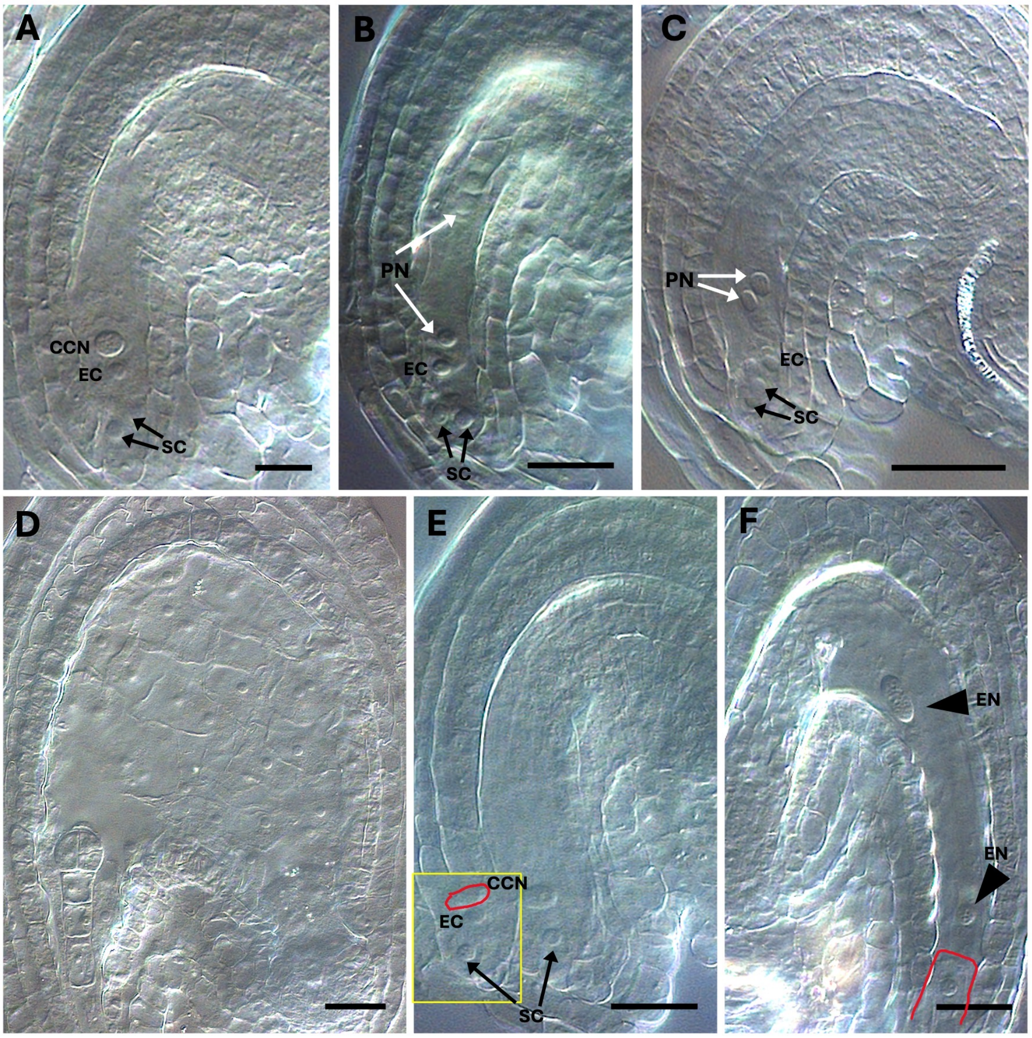

Supplementary Fig S5. Ovules and developing seeds from *ebo1-2/+* plants.

(**A**) A normal developed ovule with a central cell nucleus (CCN), one egg cell (EC), and two synergid cells (SC) in a mature *ebo1-2/+* pistil, the stamen were romove to avoid pollination.

(**B**) Ovule defects in *ebo1-2/+* samples, showing two unfused polar nucleus (white arrows) separated by the large vacuole.

(**C**) Ovule defects in *ebo1-2/+* samples, showing two polar nuclei (white arrows) got closed to each other but remained unfused.

(**D**) A normally developing seed in an *ebo1-2/+* silique at the two- or four-cell embryo stage, 2 days after pollination.

(**E**) An unfertilized ovule from the same silique as in (D), with a central cell nucleus with an irregular shape. The area within yellow frame is another focal plane showing the egg cell and another synergid cell, the red circle marks the position of central cell.

(**F**) A fertilized ovule in the same *ebo1-1/+* silique as in (D), showing two endosperm nuclei (EN) generated from the first division of fertilized central cell nucleus differing in size and shape, and the elongated zygote outlined by the red line.

CCN, central cell nucleus; EC, egg cell; SC, synergid cell; PN, polar nucleus; EN, endosperm nucleus. Scale bars = 20 μm.

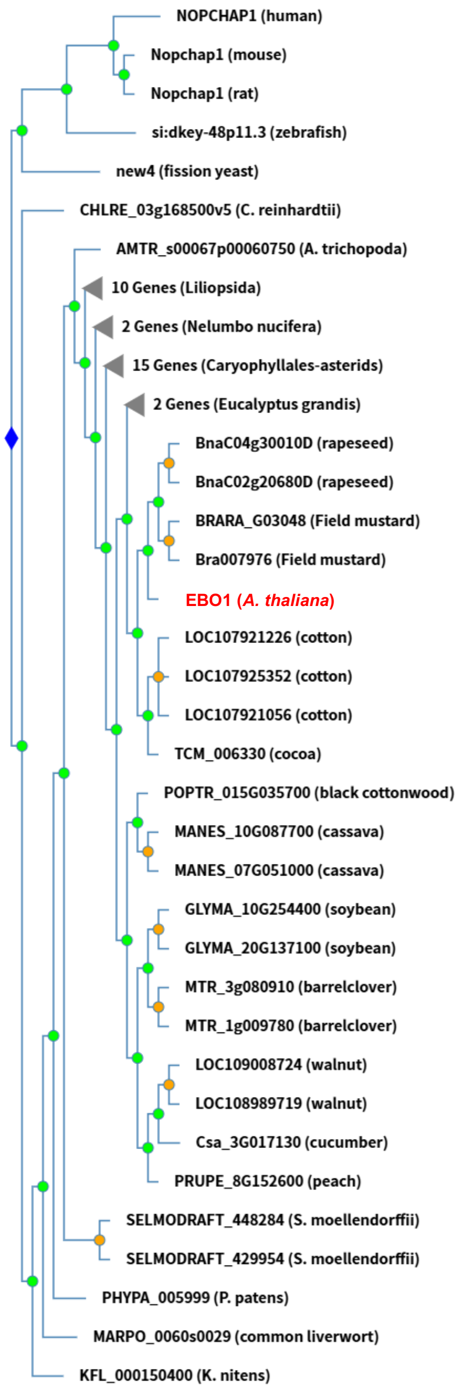

Supplementary Figure S6. The phylogenetic tree including EBO1 and NOPCHAP1. The tree was downloaded from https://phylogenes.arabidopsis.org/tree/PTHR28674.

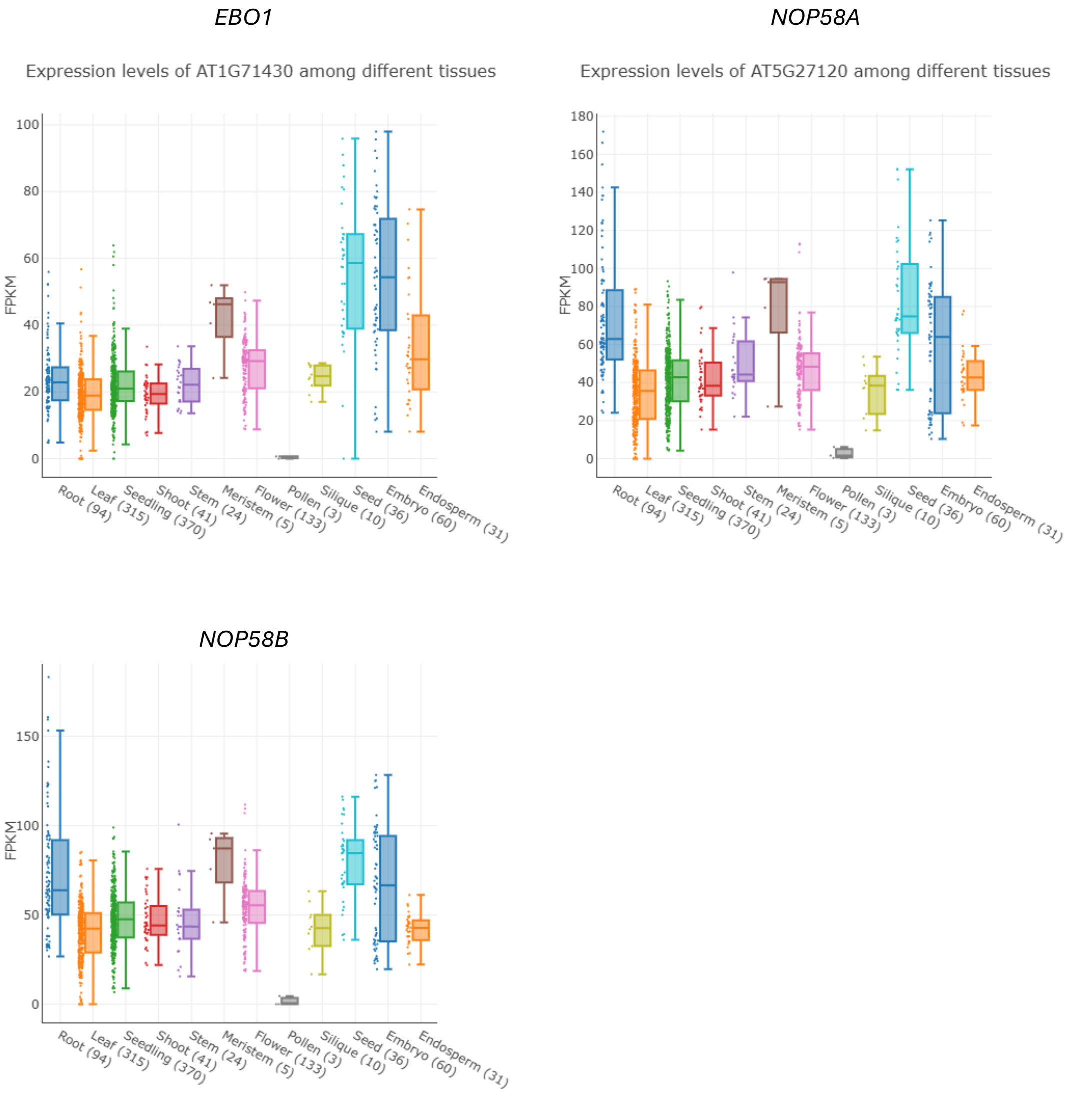

Supplementary Figure S7a. The expression pattern of EBO1, NOP58A and NOP58B. The data was downloaded from https://plantrnadb.com/athrdb/

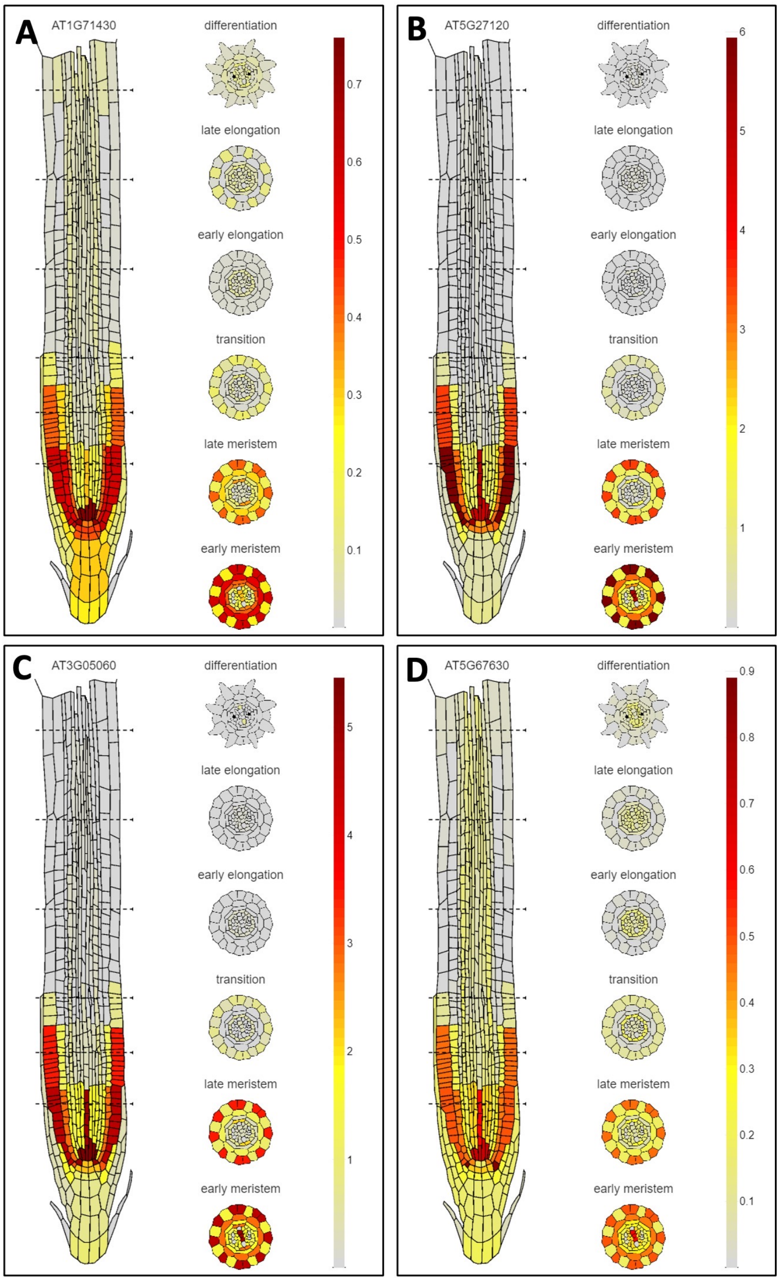

Supplementary Fig S7b. The expression profiles in root of *EBO1* and the genes coding for the candidate interactors. (A) *EBO1*; (B) *NOP58A*; (C) *NOP58B*; (D) *RUVBL2*
Data was integrated and published by RootCellAtlas (2022) https://rootcellatlas.org/

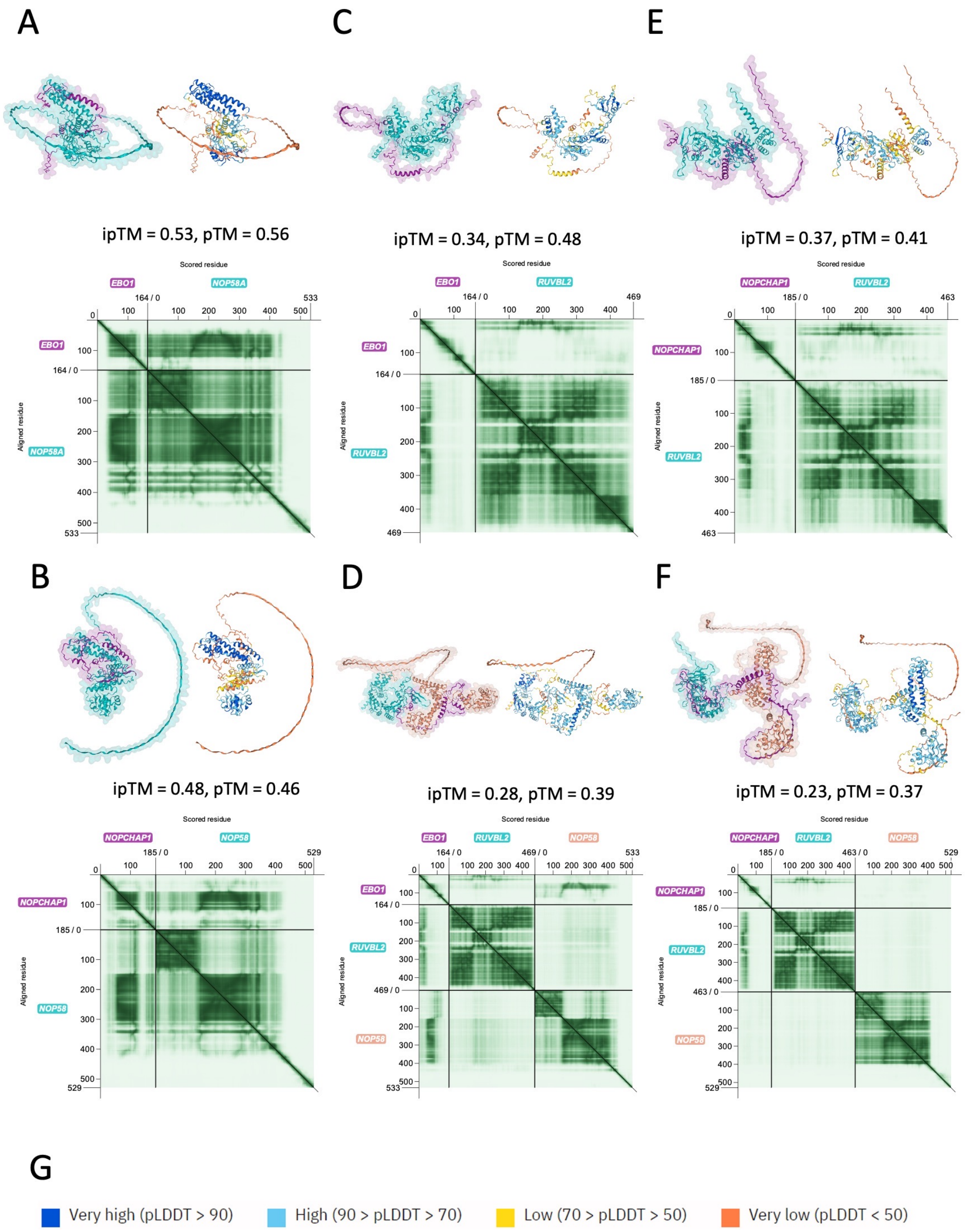

Supplementary Fig S8. Three-dimensional structural models of the complexes formed by EBO1 and interactors.
AlphaFold predicted structures of the EBO1-NOP58A dimer (A), the NOPCHAP1-NOP58 dimer (B), the EBO1-RUVBL2 dimer (C), the EBO1-RUVBL2-NOP58A trimer (D), the NOPCHAP1-RUVBL2 dimer (E) and the NOPCHAP1-RUVBL2-NOP58 trimer (F). The structure color scheme is shown by subunits (left panel) or by Per-residue measure of local confidence (pLDDT) scores (right panel). The color codes for pLDDT scores are indicated in (G). The interface predicted template modelling (ipTM) scores, and the predicted template modelling (pTM) scores are also shown. The predicted aligned error (PAE) plots provided for each predicted structure demonstrate the high confidence (dark green) and low confidence (pale green) regions for the predicted structure. The colour coding indicates the protein identity as shown in the PAE plots.

A

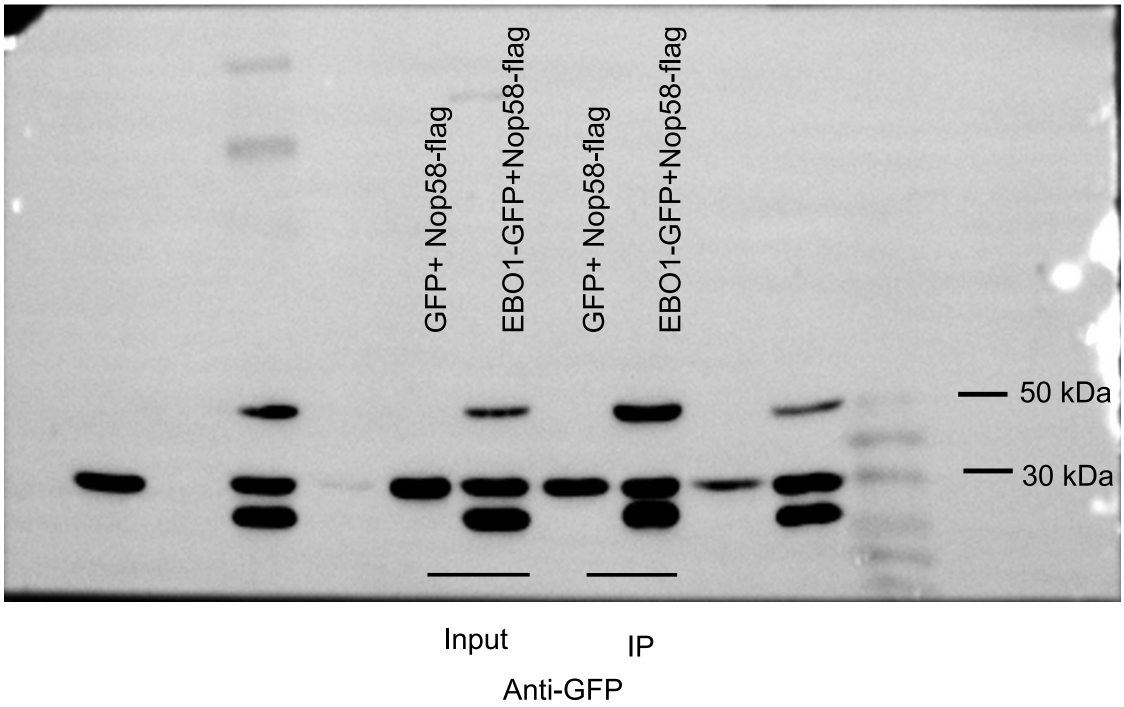

B

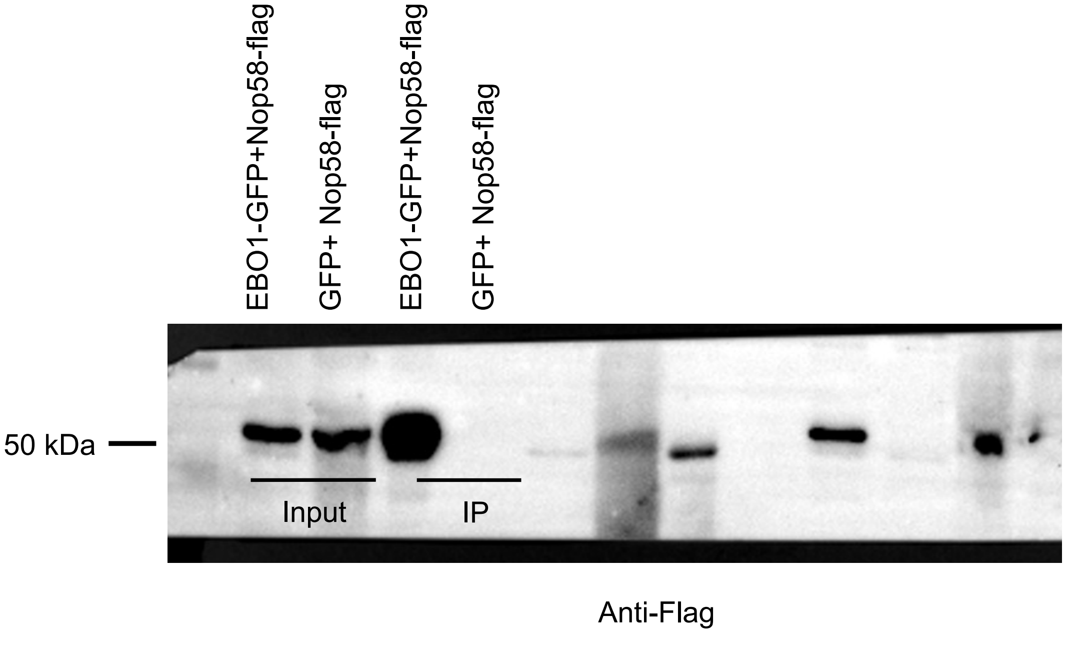

Supplementary Figure S9. The entire Western blots of (A) the EBO1-GFP and (B) Nop58-FLAG co-immunoprecipitation experiments corresponding to blots shown in the main figure 7G.

Supplementary Table S1. Common interactors identified between Arabidopsis EBO1 and human NOPCHAP1

Arabidopsis proteins AT1G71430 EBO1		Human proteins NOPCHAP1	Description
AT5G27120<br>AT3G05060	NOP58A<br>NOP58B	NOP58	Nucleolar protein 58, Core component of box C/D small nucleolar ribonucleoprotein (snoRNP) particles. Part of the ribosome small subunit (SSU) processome (Singh *et al*. 2021).
AT5G67630	RUVBL2	RUVBL1<br>RUVBL2	DNA-dependent ATPase and DNA helicase, belongs to the ATPases associated with diverse cellular activities (AAA+) protein family. Component of the NuA4 histone acetyltransferase complex which is involved in transcriptional activation. Component of the chromatin remodeling INO80 complex and a SWR1-like complex. box C/D snoRNP assembly (Puri *et al*., 2007; López-Perrote *et al*., 2020; Doyon *et al*., 2004; Obri *et al*.,2014; Jin *et al*.,2005; Chen *et al*., 2011).
AT5G61140	BRR2C	SNRNP200	U5 small nuclear ribonucleoprotein 200 kDa helicase (Bergfort *et al*., 2022; Zhang *et al*., 2019).
AT1G06220	CLO	EFTUD2	116 kDa U5 small nuclear ribonucleoprotein component (Zhang *et al*., 2017; Liu *et al*., 2006).
AT1G80070	PRP8A	PRPF8	220 kDa U5 small nuclear ribonucleoprotein specific protein (Pre-mRNA-processing-splicing factor 8) (Luo et al., 1999).
AT1G20580	SMD3B	SNRPD3	Small nuclear ribonucleoprotein Sm D3 (Sm-D3) (snRNP core protein D3) (Jurica et al., 2002).
AT4G00830	LIF2	HNRPQ	Heterogeneous nuclear ribonucleoprotein Q (hnRNP Q) (Grosset et al., 2000; Blanc et al. 2001).
AT5G53180	PTBP2	HNRNPL	Heterogeneous nuclear ribonucleoprotein L (hnRNP L) (Loh *et al*., 2017).
AT3G03960	CCT8	CCT8	T-complex protein 1 subunit (Freund *et al*., 2014)
AT5G02500	HSP70-1	HSPA1A	Heat shock 70 kDa protein
AT5G02490	HSP70-2	HSPA4	Heat shock 70 kDa protein
AT3G09440	HSP70-3	HSPA5	Heat shock 70 kDa protein
AT3G12580	HSP70-4	HSPA8	Heat shock 70 kDa protein
AT1G79920	HSP70-15	HSPH1	Heat shock 70 kDa protein
AT2G09990	RPS16	RPS16	40S ribosomal protein S16
AT1G22780	RPS18	RPS18	40S ribosomal protein S18
AT1G18080	RACK1A	RACK1	40S ribosomal subunit protein RACK1
AT4G16720	RPL15	RPL15	60S ribosomal protein L15
AT2G46280	EIF3I1	EIF3I	Eukaryotic translation initiation factor 3 subunit I
